# Whole Genome Analysis of Sugarcane Root-Associated Endophyte *Pseudomonas aeruginosa* B18—A Plant Growth-Promoting Bacterium With Antagonistic Potential Against *Sporisorium scitamineum*

**DOI:** 10.3389/fmicb.2021.628376

**Published:** 2021-02-05

**Authors:** Pratiksha Singh, Rajesh Kumar Singh, Dao-Jun Guo, Anjney Sharma, Ram Nageena Singh, Dong-Ping Li, Mukesh K. Malviya, Xiu-Peng Song, Prakash Lakshmanan, Li-Tao Yang, Yang-Rui Li

**Affiliations:** ^1^Key Laboratory of Sugarcane Biotechnology and Genetic Improvement (Guangxi), Ministry of Agriculture, Sugarcane Research Center, Chinese Academy of Agricultural Sciences, Guangxi Key Laboratory of Sugarcane Genetic Improvement, Sugarcane Research Institute, Guangxi Academy of Agricultural Sciences, Nanning, China; ^2^Guangxi Key Laboratory of Crop Genetic Improvement and Biotechnology, Nanning, China; ^3^College of Agriculture, Guangxi University, Nanning, China; ^4^AgriGenome Labs Pvt., Ltd., Kochi, India; ^5^Microbiology Institute, Guangxi Academy of Agricultural Sciences, Nanning, China; ^6^Interdisciplinary Research Center for Agriculture Green Development in Yangtze River Basin (CAGD), College of Resources and Environment, Southwest University, Chongqing, China; ^7^Queensland Alliance for Agriculture and Food Innovation, The University of Queensland, St Lucia, QLD, Australia

**Keywords:** biocontrol, biotic stress, endophyte, genome sequence, plant growth promotion, *Pseudomonas aeruginosa*, sugarcane

## Abstract

Sugarcane smut is a significant fungal disease that causes a major loss in sugar yield and quality. In this study, we isolated an endophytic strain B18 from a sugarcane root, which showed plant growth-promotion, hydrolytic enzyme production, antifungal activity against sugarcane pathogens (*Sporisorium scitamineum, Ceratocystis paradoxa, Fusarium verticillioides*), and the presence of *nifH*, *acdS*, and antibiotic genes (*hcn, prn*, and *phCA*) under *in vitro* conditions. BIOLOG^(R)^ phenotypic profiling of B18 established its ability to use various carbon and nitrogen sources and tolerate a range of pH and osmotic and temperature stresses. Whole-genome analysis of B18, identified as *Pseudomonas aeruginosa*, showed that it consists of a single circular chromosome of 6,490,014 bp with 66.33% GC content. Genome annotation has identified 5,919 protein-coding genes, and 65 tRNA, and 12 rRNA genes. The *P. aeruginosa* B18 genome encodes genes related to ethylene, nitrogen (*nifU*, *norBCDERQ*, *gltBDPS*, and *aatJMPQ*), and phosphate (*pstABCS* and *phoBDHRU*) metabolism and produce indole-3-acetic acid and siderophores. This also includes genes encoding hydrolases and oxidoreductases, those associated with biocontrol mechanisms (*hcnABC*, *phzA_B*, *phzDEFGMS*, and *pchA*), colonization (*minCDE* and *lysC*), and biofilm formation (*efp*, *hfq*, *flgBCDEFGHI*, and *motAB*), and those associated with metabolism of secondary metabolites. Collectively, these results suggest a role for *P. aeruginosa* B18 in plant growth enhancement and biocontrol mechanisms. The *P. aeruginosa* B18 strain was found to be an efficient colonizer in sugarcane; it can improve growth through modulation of plant hormone production and enhanced host-plant resistance to smut pathogen *S. scitamineum* in a smut-susceptible sugarcane variety (Yacheng71-374). These biocontrol and plant growth promotion properties of *P. aeruginosa* B18 area are discussed in this report.

## Introduction

Sugarcane (*Saccharum* spp. interspecific hybrids) is the most prominent economic crop in tropical and sub-tropical countries and the world’s main source of sugar. Sugar is a major agricultural commodity, and it is also used for producing ethanol and other by-products (FAOSTAT, 2020). In China, sugarcane is a key agricultural crop, and it contributes to about 90% of sugar production nationally. Besides a limited water supply, the occurrence of various diseases and insect pests and the overuse of fertilization also adversely affect Chinese sugarcane production (Li and Yang, 2015). Presently, more than 60 sugarcane diseases have been reported in China (Huang and Li, 2016). Among them, sugarcane smut, caused by the fungal pathogen *Sporisorium scitamineum*, is a major disease. It is wide-spread in all the major sugarcane production areas in China, such as Guangxi, Yunnan, Guangdong, and Hainan. Sugarcane smut disease control currently relies primarily on breeding resistant cultivars (Shen et al., 2014), which is constrained by long, expensive breeding procedures and limited success (Liu et al., 2017). The disease can be managed by chemical fungicides (Bhuiyan et al., 2012) but is neither advised nor practiced to prevent environmental degradation (Pandin et al., 2017).

Biological control agents (BCAs) provide cost-effective, environmentally friendly pest and pathogen control in many crops, including sugarcane (Li et al., 2018; Jayakumar et al., 2019). The method of achieving biological control is complex and cannot be effective in certain production conditions. A range of non-pathogenic microbial species has the potential to trigger induced systemic resistance (ISR) by producing elicitors that trigger immune responses in plants (Cawoy et al., 2014). Plant growth-promoting endophytic bacteria (PGPEB) are microorganisms that reside and colonize inside plant tissue have also been explored widely for host plant resistance to pathogen attacks (Choudhary et al., 2007; Kumar et al., 2017). These bacteria simply enter the plant roots through various means and promote plant growth through different mechanisms, such as plant growth regulators, phosphate solubilization, nitrogen-fixation, ethylene metabolism, and indirect disease resistance mechanisms by antimicrobial metabolites or siderophores that suppress pathogenic microbes (Sun et al., 2009; Olanrewaju et al., 2017). Hence, the use of PGPEB is receiving a renewed interest as a green alternative to agrochemicals for sustainable agriculture.

In plant-associated environments, *Pseudomonas* organisms are ubiquitous and play a significant role in the natural defense of plants against pathogens (Mendes et al., 2007). Bacteria belonging to the genus *Pseudomonas* protect plants by direct competition with or being antagonistic to pathogens (Haas and Défago, 2005; Bakker et al., 2007). Many *Pseudomonas* species, especially *P. fluorescens*, *P. putida*, *P. chlororaphis*, and *P. syringe*, are well recognized for their ability to stimulate plant growth and control a range of plant pathogens (Raaijmakers and Mazzola, 2012; Li et al., 2017). Some of the earlier literature reported the isolation of *Pseudomonas* strains from sugarcane: *Pseudomonas* spp., *P. aeruginosa*, *P. aurantiaca*, *P. fluorescens*, *P. putida*, *P. reactans*, *P. monteilii, P. plecoglossicida, P. entomophila, P. koreensis*, and *P. mosselii* (Viswanathana and Samiyappan, 2002; Viswanathan et al., 2003; Mendes et al., 2007; Mehnaz et al., 2009, 2010; Magnani et al., 2010; Li et al., 2017). *P. aeruginosa* strains are touted to be an important tool for disease management programs in tropical countries owing to their biocontrol effectiveness against several pathogens (Kumar et al., 2013). However, *P. aeruginosa* is often referred to as an opportunistic pathogen that colonizes various groups of organisms, and a comprehensive understanding of *P. aeruginosa* strains is limited.

In this manuscript, we focused on the endophytic *P. aeruginosa* B18 strain isolated from sugarcane roots, which promotes sugarcane growth and tolerance to smut. Whole-genome analysis of this strain will provide opportunities to identify genes involved in plant growth promotion (PGP) and biocontrol of pathogens. To the best of our knowledge, this is the first study of the whole-genome analysis of endophyte *P. aeruginosa* B18 isolated from sugarcane root. Hence, we aimed to study the *P. aeruginosa* B18 endophyte isolated from sugarcane root in relation to (i) plant-growth-promoting and antifungal activities, (ii) functional genes, (iii) production of cell wall degrading enzymes, (iv) metabolic profiling, (v) host colonization pattern (confocal laser scanning microscopy-CLSM and scanning electron microscopy-SEM), (vi) whole-genome analysis, and (v) improvement of sugarcane growth under smut pathogen stress in the greenhouse condition.

## Materials and Methods

### Isolation of Strain B18 Endophytic Strain

Sugarcane plant samples were obtained from the field of Sugarcane Research Institute, Guangxi Academy of Agricultural Sciences, Nanning, China (latitude 22°50′ N, longitude 108°14′ E, and elevation 70 m). Endophytic strain B18 was isolated from the roots of the sugarcane plant by using Ashby’s glucose agar medium according to the method of Dobereiner et al. (1993) (Supplementary Table 1). The isolated strain was maintained in glycerol solution (25%) at −20°C.

### *In vitro* Screening for Biocontrol, PGP Traits, Hydrolytic Enzymes Production, and Abiotic Stress Tolerance

The antifungal activity of strain B18 was tested against three sugarcane pathogens (*S. scitamineum, Ceratocystis paradoxa, Fusarium verticillioides*) by dual culture plate and agar well diffusion methods according to the procedure of Singh et al. (2013). For dual culture plate assay, the strain was streaked 3 cm in the gap opposite to fungal pathogens spotted at the central point of the potato dextrose agar and nutrient agar (1:1) plate and kept at 26 ± 2°C for 5–7 days. The plate included with only the fungal disk served as the control, and the percentage of inhibition was measured. For the agar well diffusion method, mycelium or spores of selected pathogens were mixed in 10 mL of autoclaved distilled water, and 0.1 mL suspension was spread on petri dishes comprising PDA (Supplementary Table 1). Wells (5 mm diameter) were prepared with cork borer into the agar medium and sealed with agarose (0.2%) before being filled with 100 μL of cell-free culture. The un-inoculated medium was taken as a control and incubated at 26 ± 2°C for 5–7 days.

To study PGP traits, strain B18 was grown in Luria Bertani (LB) medium for 36–48 h at 32 ± 2°C in an orbital shaker (120 rpm) for inoculum preparation (Supplementary Table 1). The PGP abilities of strain B18 were evaluated by standard methods that determine the qualitative and quantitative estimation of siderophore (Schwyn and Neilands, 1987; Hu and Xu, 2011), ammonia production (Dey et al., 2004; Goswami et al., 2014), and P- solubilization (Pikovskaya, 1948; Mehta and Nautiyal, 2001). Hydrogen cyanide (HCN) production was determined by the qualitative method (Lorck, 1948). Indole-3-acetic acid production was analyzed spectrophotometrically with the procedure explained by Glickmann and Dessaux (1995).

Microorganisms play a direct role in the inhibition of fungal pathogen growth through the synthesis of cell-wall-degrading enzymes. As a result, strain B18 supernatant was used for the estimation of four enzymes activities, *i.e.*, β-1, 3 glucanase (product no. MM91504O1), chitinase (product no. MM1062O1), cellulase (product no. MM91502O1), and protease (product no. MM1206O1), by enzyme-linked immune sorbent assays (ELISA) using commercially available ELISA kits (Wuhan Colorful Gene Biological Technology Co., Ltd., Wuhan, China) and following manufacturer’s instructions. Strain B18 was streaked on LB medium and kept at 32 ± 2°C for 24–48 h. A single bacterial colony was transferred to LB broth medium (10 mL) and incubated for 24–48 h in an orbital shaker (180 rpm) at 32 ± 2°C before being centrifuged at 12,000 rpm for 5 min to get the supernatant. The whole extraction process was completed at 4°C.

B18 bacterial culture (0.1 mL) was inoculated in LB broth medium (5 mL) and incubated at 20, 25, 30, 35, 40, and 45°C for 36 h at 120 rpm in an orbital shaker to determine its temperature tolerance capacity. The pH tolerance test was performed by growing 0.1 mL of B18 bacterial suspension in 5 mL of LB broth medium adjusted to different pHs (5–10) followed by incubation at 32 ± 2°C for 36 h. NaCl tolerance was examined by growing B18 isolate with different NaCl concentrations (7–12%) at the optimum pH (7) followed by incubation for 36 h at 32 ± 2°C. After incubation, B18 growth was measured at 600 nm using a spectrophotometer, and the un-inoculated medium was applied as a blank.

### 1-Aminocyclopropane-1-Carboxylate (ACC) Deaminase and Acetylene Reduction Assay (ARA)

The 1-Aminocyclopropane-1-carboxylate deaminase activity of the B18 strain was examined with nitrogen-free Dworkin and Foster (DF) salts minimal medium (Jacobson et al., 1994) (Supplementary Table 1). Medium devoid of ACC was applied as a negative control, and medium with (0.2% w/v) ammonium sulfate [(NH_4_)_2_SO_4_] or (3 mM) ACC was used as a positive control. The growth of B18 was observed at 32 ± 2°C after 3–5 days of incubation. ACC deaminase activity was quantified as described earlier Honma and Shimomura method (Honma and Shimomura, 1978). The nitrogen-fixation potential of the B18 strain was studied by the ARA method according to the procedure of Hardy et al. (1968).

### Amplification and Sequencing of *acdS*, *nifH*, and Antibiotic Genes

Strain B18, DNA was used as a template for amplifying all five selected genes (*acdS*, *nifH*, *hcn*, *phCA*, and *prn*). Amplification of *acdS* and *nifH* genes was done by using degenerate primers following the PCR conditions reported earlier (Poly et al., 2001; Li et al., 2011; Supplementary Table 2). *hcn* gene amplification was completed with HCNF and HCNR primers according to Ramette et al. (2003) (Supplementary Table 2). Antibiotic genes, *i.e*., *phCA* and *prn* were amplified by using the primers given in Supplementary Table 2 and followed the PCR conditions detailed in Raaijmakers et al. (1997) and Souza and Raaijmakers (2003). All purified PCR products were sequenced (Sangon Biotech, Shanghai, China). Genes were identified using the NCBI GenBank database.

### BIOLOG^(R)^ Phenotypic Characterization

Phenotypic profiling of B18 isolate was performed on GENIII, PM3B, PM9, and PM10 Biolog microplates; a tetrazolium-based growth test revealed by Biolog Incorporated (Biolog, Inc., Hayward, CA, United States) was used to analyze carbon and nitrogen substrate uses along with osmotic and pH tolerance (Bochner, 2009). A total of 96 wells were used in the BIOLOG Micro-ArrayTM plates, and each well includes a separate formulation to detect the use of substrate or stress sensitivity. GENIII and PM3B plates were used to identify strains for their ability to utilize different sources of carbon and nitrogen, whereas PM9 and PM10 plates were used for high salt concentrations and extreme pH microbial tolerance screening. The inoculum for microplates was prepared as described earlier (Li et al., 2017), and the inoculated microplates were kept at 32 ± 2°C for 72 h to develop tetrazolium color. The reading was taken with preset BIOLOG^(R)^ Micro-Station Reader following the manufacturer’s instructions. The bacteria growth was calculated with optical density measurements at 590 nm after 72 h of incubation. All data are presented using a heatmap created by Heml1.0 software (Deng et al., 2014).

### Colonization of B18 in Sugarcane

The pPROBE-pTetr-TT plasmid containing the green fluorescent protein (GFP) gene was obtained from Agriculture College, Guangxi University, Nanning, China, and a bacterial strain sample was prepared as described previously (Singh et al., 2020b). Micro-propagated sugarcane plants were examined by confocal laser scanning microscopy (CLSM) (Leica DMI 6000, Germany) 96 h after bacterial inoculation. The scanning electron microscopy (SEM) (Hitachi model SU8100) was used to confirm the morphology and colonization of B18 stain in sugarcane as described by Singh et al. (2013).

### DNA Isolation, Library Creation, and Genome Sequencing

Based on *in vitro* multi-functional activities of strain B18, we studied the genome analysis of this strain to completely understand the characteristics of this bacterium. Genomic DNA of strain B18 was extracted with Wizard^®^ Genomic DNA Purification Kit (Promega) as per the manufacturer’s guidelines. DNA concentration and quality were assessed by TBS-380 fluorometer (Turner BioSystems Inc., Sunnyvale, CA, United States), and good quality DNA (OD260/280 = 1.8∼2.0, > 20 μg) was used for additional analysis. The genome was sequenced by Oxford-Nanopore methods. The 15 μg purified DNA was rotated in a Covaris G-TUBE (Covaris, MA, United States) for 60 s at 6,000 rpm with an Eppendorf 5424 centrifuge (Eppendorf, NY, United States) for Nanopore sequencing. DNA fragments were purified, end-repaired, and ligated through SMRTbell sequencing adapters as per the manufacturer’s procedure (Pacific Biosciences, CA, United States). The ensuing sequencing library was purified three times with 0.45 × volumes of Agencourt AMPureXP beads (Beckman Coulter Genomics, MA, United States) as per the manufacturer’s commands. Subsequently, a ∼10 kb insert library was organized and sequenced on one SMRT cell by standard procedures. After obtaining the qualified genomic DNA, the large size fraction was selected by an automated DNA size selection system (Blue Pippin, Sage Science); the DNA was then treated with the damage repair and end-repair/dA tailing module. After purification, adapter ligation was performed by using a ligation sequencing kit (NBD103 and NBD114, Oxford Nanopore Technologies). Finally, the DNA library was quantified by Qubit (Thermo fisher TEchnlogies). A certain concentration and volume of the DNA library were loaded into a 1 flow cell, which was then transferred to Nanopore PromethION sequencer (Oxford Nanopore Technologies) for real-time single-molecule sequencing.

### Genome Assembly, Annotation, and Prediction of Genes in B18 Genome

The raw sequence data generated from Illumina and Nanopore sequencing were utilized for bioinformatics investigation, and the entire assessments were completed with the free online Majorbio Cloud Platform^1^ (Shanghai Majorbio Co., Ltd.). The whole-genome sequence was assembled by using both Illumina and Nanopore quality reads. For quality trimming, a value data statistic was used, from which the low-value information can be eliminated to form clean reads. The reads were then assembled into contigs by the hierarchical genome assembly method (HGAP) and canu (Koren et al., 2017). The final step was completed and finished manually, generating a whole-genome with seamless chromosome, and the final error correction of the Nanopore assembly results was done with the Illumina reads by Pilon.

Prediction of coding sequence (CDS) was finished with Glimmer version 3.02 followed by annotation from different databases, *i.e*., Pfam, NR, Swiss-Prot, Clusters of Orthologous Groups (COG), Gene Ontology (GO), and Kyoto Encyclopedia of Genes and Genomes (KEGG) databases (Delcher et al., 2007) with Basic Local Alignment Search Tool (BLAST), HMMER, and Diamond sequence alignment tools. Prediction of tRNA and rRNA was done by tRNA-scan-SE (v1.2.1) (Borodovsky and Mcininch, 1993) and Barrnap. Antismash software was used for predicting secondary metabolite genes. Briefly, each set of query proteins were aligned through the databases, and annotations of best-coordinated subjects (*e*-value < 10^–5^) were made for the annotation of genes. Genome annotation files (genome assembly.fasta and genome annotation.gff) were uploaded to the CGviewer server (Grant and Stothard, 2008) to prepare a Circular genome map of strain B18.

### Phylogenetic Analysis of Strain B18

The 16S rRNA gene sequences were obtained from the assembled genome and used for BLAST (BLASTn) study against the ribosomal database (rRNA_type strains/16S_ribosomal_RNA) from NCBI for the strain B18 identification. A total of 74 16S rRNA gene sequences were downloaded from the *Pseudomonas* species and used for the phylogeny study. The bootstrap study was conducted using 1000 pseudo-replication by the Felsenstein method (Felsenstein, 1985). The tree was drawn to scale through branch lengths in similar units as those of the evolutionary distances applied to complete the phylogenetic tree. The evolutionary distances were calculated by the Maximum Composite Likelihood procedure (Tamura et al., 2004). For every sequence pair (pairwise deletion option), all unclear positions were eliminated. The final dataset had a total of 1556 places. Evolutionary analyses were completed in MEGA X (Kumar et al., 2018).

### Comparative Genome Analysis

Comparative genome analysis was performed by the RAST analysis server (KbaseGenomes4.1)v to understand the genome features of strain B18. Average Nucleotide Identity (ANI) analysis of strain B18 was achieved by FastANI (Jain et al., 2018). FastANI is built to complete genome sequences for rapid alignment-free computation of complete-genome ANI and facilitates the comparison of whole and draft genome assemblies in pairs. The complete genome of strain B18 and two *Pseudomonas* strains (*Pseudomonas aeruginosa* PA01 and *Pseudomonas* spp. PL10) existing in the NCBI database were used for this study.

### Greenhouse Experiment

The ability of strain B18 to promote sugarcane plant growth under smut disease was evaluated using a pot experiment with four treatments (described below) (i) YC, (ii) B18, (iii) SP, and (iv) B18 + SP, each with five replicates in a greenhouse. The soil used for the experiment was autoclaved for 30 min (at 121°C) thrice. The trial was conducted with smut susceptible sugarcane variety Yacheng71-374, obtained from the Guangxi Academy of Agricultural Sciences, Nanning, China. Healthy sugarcane seedlings (45 days old) were used for the experiment, and they were washed with tap water to eliminate soil particles adhered to the surface of the plants prior to pathogen inoculation. In order to prepare the inoculum, *S. scitamineum* haploid strains (MAT-1 and MAT-2) were grown in 100 mL YEPS liquid broth at 26 ± 2°C for 2–3 days in an incubator shaker (120 rpm) (Supplementary Table 3). Strain B18 was grown in 100 mL LB broth at 32 ± 2°C with shaking at 120 rpm for 24–48 h. At the end of incubation, bacteria were collected by centrifugation at 6000 rpm for 5 min, washed twice with autoclaved distilled water, and re-suspended in sterile distilled water (OD. 600 ∼ 5.0).

For treatment (YC), Yacheng71-374 seedlings were injected with 1 mL of autoclaved sterile water (about four leaves) at the stem base. Similarly, for treatment B18 and SP, seedlings were inoculated with 1 mL of strain B18 and a 1:1 mixture of MAT1 and MAT2 suspension, respectively. For B18 + SP treatment, seedlings were treated with 1 mL of bacterial suspension and 1 mL of the fungal mixture (MAT1 and MAT2; 1:1). Each treatment had five replicates, and the inoculation was performed at 30 ± 2°C and > 80% relative humidity. Inoculated seedlings were moved into plastic pots (30 cm in diameter, 40 cm in-depth, and three plants per pot) containing 15 kg of soil and sand mixture (3:1 w/w). The sugarcane plantlets were not supplied with any chemical fertilizers and watered on alternate days.

### Physiological Parameters, Pathogen Defense-Related Enzymes, Phytohormones, and qRT-PCR

Sugarcane plant samples were collected after 4 weeks following treatment, and plant height, fresh shoot weight, fresh root weight, leaf area, chlorophyll content, photosynthesis, transpiration rate, and stomatal conductance were measured.

Sugarcane leaf samples were collected 4 weeks after treatment, powdered with liquid nitrogen, and used for the estimation of pathogen defense-related enzymes such as peroxidase (POD), catalase (CAT), superoxide dismutase (SOD), β-1,4-endoglucanase, and chitinase along with phytohormones, including gibberellins (GA_3_), ethylene (ETH), abscisic acid (ABA), and indole-3-acetic acid (IAA) by plant enzyme-linked immune sorbent assays (ELISA) kit (Wuhan Colorful Gene Biological Technology Co., Ltd., China) following the manufacturer’s instructions.

The expression pattern of *SuCHI*, *SuGLU*, *SuCAT*, and *SuSOD* genes was examined in the sugarcane variety Yacheng71-374 at the end of 4 weeks of treatment using glyceraldehyde-3-phosphate dehydrogenase (GAPDH) as a reference gene (Niu et al., 2015). Leaf samples (100 mg) were collected from all four treatments, *i.e*., (i) YC, (ii) B18, (iii) SP, and (iv) B18 + SP, and total RNA was isolated using a trizol reagent (Tiangen, Beijing, China) as per manufacturer’s protocol. DNA impurity of RNA samples was eliminated using DNase I (Promega, Fitchburg, WI, United States), and the purity and yield of isolated RNA were checked with the Nano photometer (Implen-3780, CA, United States). For these RNA samples, single-strand cDNA was synthesized with the Prime-ScriptTM RT Reagent Kit, 1 μg of total RNA was used (TaKaRa, China). Primer sequences used in this study are presented in Supplementary Table 4. The specificity of primers was checked by melt curve analysis. Relative gene expression was measured by the expression level of the treated analysis minus control expression level (treatment I: YC no microorganism) following 2^–ΔΔ*ct*^ method (Livak and Schmittgen, 2001). All qRT-PCR studies were conducted with a Real-Time PCR Detection System with five replicates (Bio-Rad, CA, United States) in SYBR Premix Ex Tap^TM^ II (TaKaRa, Japan) following the PCR conditions of Singh et al. (2018, 2019).

### Statistical Analysis

Experimental data were subjected to analysis of variance (ANOVA) following by Duncan’s multiple range test (DMRT) for testing mean differences. For all mean values, standard errors were calculated, and the significance level at *p* ≤ 0.05 was determined. All PGP and biochemical activities were done in three replicates and represented as mean values.

## Results

### PGP Traits, Biocontrol, and Abiotic Stress Tolerance Properties of Strain B18

**FIGURE 1:**
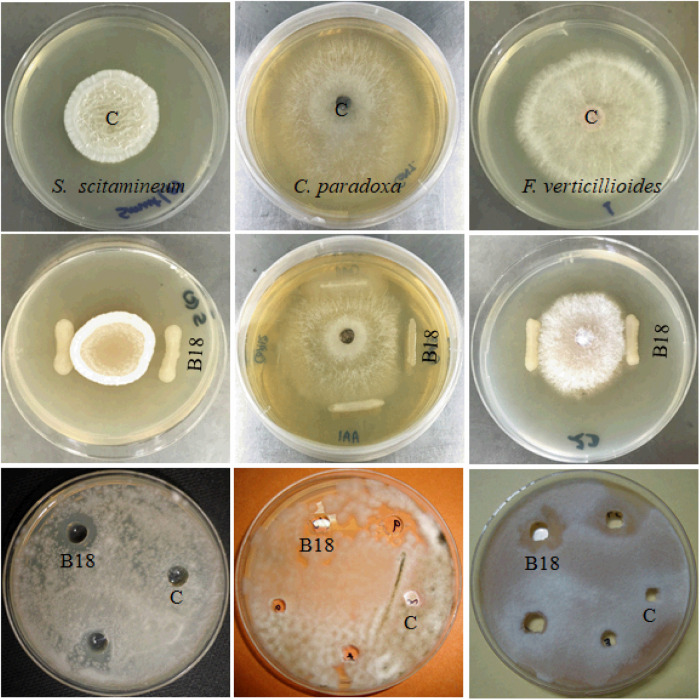
Antifungal activity of strain B18 against *Sporisorium scitamineum*, *Ceratocystis paradoxa*, and *Fusarium verticillioides* sugarcane pathogens. The first row shows control plates, the second row shows growth inhibition of pathogens by strain B18 in dual culture plate assay, and the third row shows the agar well diffusion method. C (control).

Endophytic bacteria B18 was initially screened to examine its antifungal activity against three sugarcane pathogens (*S. scitamineum, C. paradoxa, F. verticillioides*), and it exhibited strong activity against *S. scitamineum* and moderate activity against *C. paradoxa* and *F. verticilliodes* (Figure 1 and Table 1). The strain B18 produced hydrolytic enzymes, such as cellulase (363.37 ± 5.37 IU mL^–1^), glucanase (732.69 ± 10.84 IU mL^–1^), protease (131.70 ± 1.95 IU mL^–1^), and chitinase (453.12 ± 6.70), under *in vitro* conditions (Table 1). It formed an orange halo zone on medium chrome azurol S agar and clear zone formation on Pikovskaya’s agar media, indicating siderophore production (68.26 ± 1.42%) and phosphate solubilization (95.2 ± 1.21 μg mL^–1^) capacity, respectively. It also showed strong ammonia (4.42 ± 0.21 μmoL mL^–1^) and moderate hydrogen cyanide (HCN) production tests (Table 1).

**TABLE 1:** Functional characteristics of *Pseudomonas aeruginosa* B18 isolated from sugarcane root.

Parameters	*P. aeruginosa* B18
**Antifungal Activity**	

*Sporisorium scitamineum*	+ ++
*Ceratocystis paradoxa*	+ +
*Fusarium verticillioides*	+ +

**Plant Growth Promoting Traits**	

Siderophore (%)	68.26 ± 1.42
Phosphate (μg mL^–1^)	95.2 ± 1.21
Ammonia (μmoL mL^–1^)	4.42 ± 0.21
ACC	+
HCN	+ +

**Abiotic Stress Tolerance**	

pH	5.0-10.0
Temperature (°C)	20-45
NaCl (%)	7-12

**Indole-3-acetic acid (μg mL**^–^**^1^)**	

Absence of Tryptophan	97.96 ± 1.33
Presence of Tryptophan (0.5%)	144.93 ± 2.14
Presence of Tryptophan (1.0%)	159.38 ± 2.36

**Hydrolytic Enzymes (IU mL**^–^**^1^)**	

Cellulase	363.37 ± 5.37
Glucanase	732.69 ± 10.84
Protease	131.70 ± 1.95
Chitinase	453.12 ± 6.70
**ACC** (nmoL α-ketobutyrate mg^–1^ h^–1^)	446.22 ± 6.60
**ARA** (nmoL C_2_H_2_ mg protein h^–1^)	11.38 ± 0.17

*(+) = Low activity; (+ +) = Moderate activity; (+ + +) = Strong activity; (−) = No activity.*

Indole-3-acetic acid (IAA) production is a main characteristic of PGPEB, and IAA production of B18 isolate was 144.93 ± 2.14 μg mL^–1^ and 159.38 ± 2.36 μg mL^–1^ in medium containing 0.5 and 1% tryptophan, respectively, and 97.96 ± 1.33 μg mL^–1^ in medium devoid of tryptophan (Table 1). After 36–48 h incubation at 32 ± 2°C, strain B18 consumed 3 mM ACC as the sole source of nitrogen in DF-ACC medium, and the estimated ACCD activity was 446.22 ± 6.60 nmol α-ketobutyrate mg^–1^ h^–1^, along with nitrogen fixating capacity (11.38 ± 0.17 nmol C_2_H_2_ mg protein h^–1^) measured using ARA (Table 1). B18 grew in a pH range of 5.0–10.0 (optimum pH 7.0) and 20–45°C temperature (optimum 35°C), as well as in the presence of 7–12% NaCl (optimum 7%, *w*/*v*) (Supplementary Table 3), indicating its versatility.

### BIOLOG^(R)^ Metabolic Profiling of Strain B18

**FIGURE 2:**
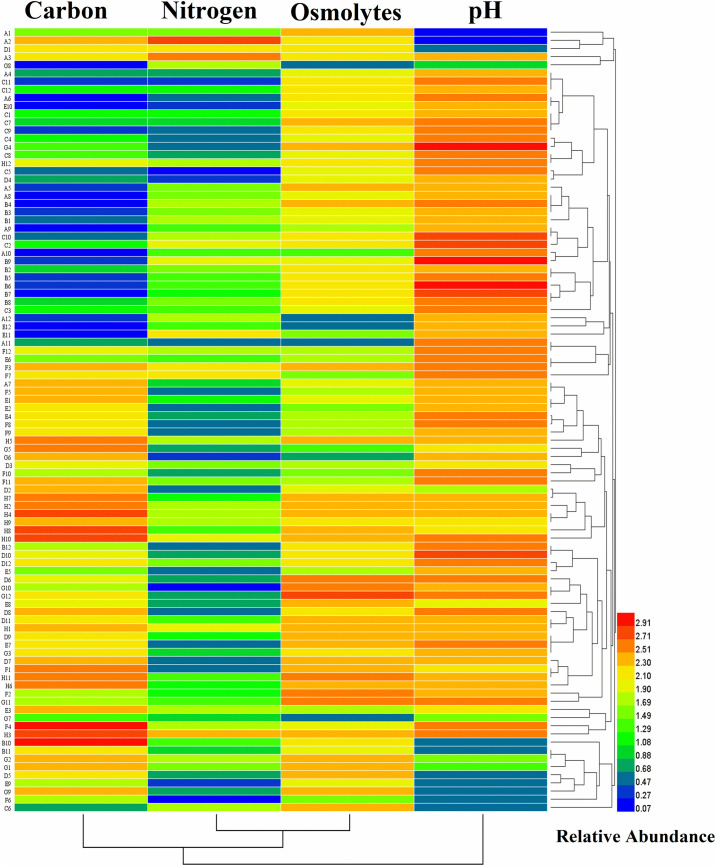
Heatmap plot representing the growth of *Pseudomonas aeruginosa* B18 as colored graphical form with relative abundance in every 95 different substrates of GNIII, PM3B PM9, and PM10 Biolog plates.

Biolog technique was applied to distinguish the physiological, biochemical, and chemical sensitivity of microorganisms based on the substrate used and assessing changes in microbial functional diversity related to other traditional bacterial cultivation approaches. The metabolic properties of an organism may lead to a particular niche adaptation, such as soil and plant tissues, which have made it possible for bacteria to respond to various environments. Therefore, we examined the metabolic abilities of strain B18 by the assimilation and tolerance of numerous carbon and nitrogen compounds to osmotic and pH stresses with GNIII, PM3B, PM9, and PM10 Biolog microplates. The heatmap shows that B18 growth was visibly distinguished in all 96 different substrates of Biolog plates (GNIII, PM3B PM9, and PM10) as colored graphical form with a relative abundance of 0.07–2.91. This is based on the utilization and tolerance of various substrates of B18 to pH and osmotic stresses (Figure 2). All 96 substrates found in the four BIOLOG plates selected are listed in Supplementary Table 4. These results established the capacity of B18’ s tolerance to environmental stresses and the consumption of varied nutrients.

### Colonization of B18 in Sugarcane

**FIGURE 3:**
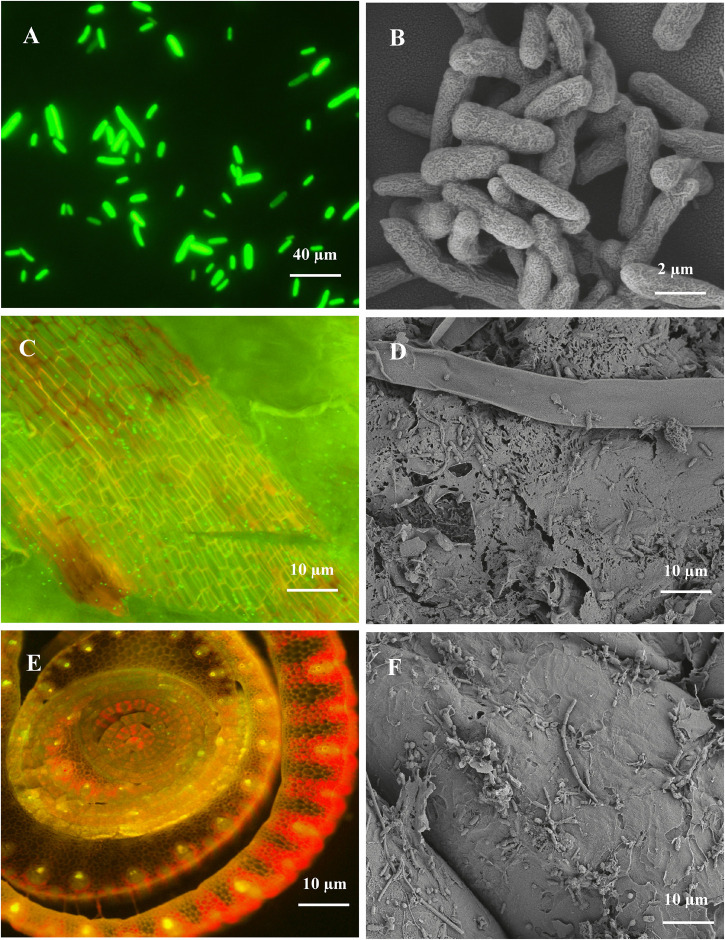
CLSM and SEM micrographs images showing morphology and colonization of *Pseudomonas aeruginosa* B18 in sugarcane variety (Yacheng71-374). **(A,B)** is rod-shaped morphology of B18 strain, **(C,D)** is the colonization of B18 in stem tissue of sugarcane, and **(E,F)** is the colonization of B18 on the root surface of sugarcane. CLSM images confirming inoculated GFP tagged B18 strain as green dots in sugarcane tissues.

The colonization ability of endophytic bacteria on plant roots is essential for disease management and plant growth improvement. In this study, we observed the colonization pattern of endophytic strain B18 on sugarcane variety Yacheng71-374 by using CLSM and SEM (Figure 3). The strain was characterized as a Gram-negative, motile, non-spore-forming, and rod-shaped bacterium (Figures 3A,B). The GFP-tagged B18 bacteria colonized in sugarcane plants were detected after 3 days of inoculation and revealed colonization as a green spot in stem and root tissues of the plant (Figures 3C–F).

### Genome Characteristics of *P. aeruginosa* B18

**FIGURE 4:**
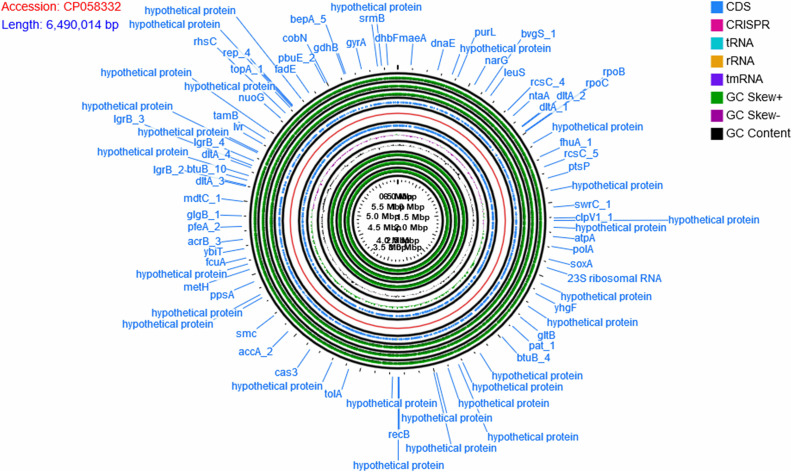
The circular map of *Pseudomonas aeruginosa* B18 genome showing CDS genes (annotated with COG and KEGG), ORFs, GC content, and GC skew (+) and GC Skew (–). Circular genome map divided into two parts by genome backbone (red color). The positive strand of the genome showed genes (CDS) in blue color followed by outer three layers showed ORFs (all three reading frames on the positive strand, in green color). The first inner circle after genome backbone showed genes (CDS) in blue color, followed by three circles of GC skew (+) in green color, GC skew (–) in purple color, and GC content in black color. The inner-most three circles belong to all three ORFs on the negative (reverse).

**FIGURE 5:**
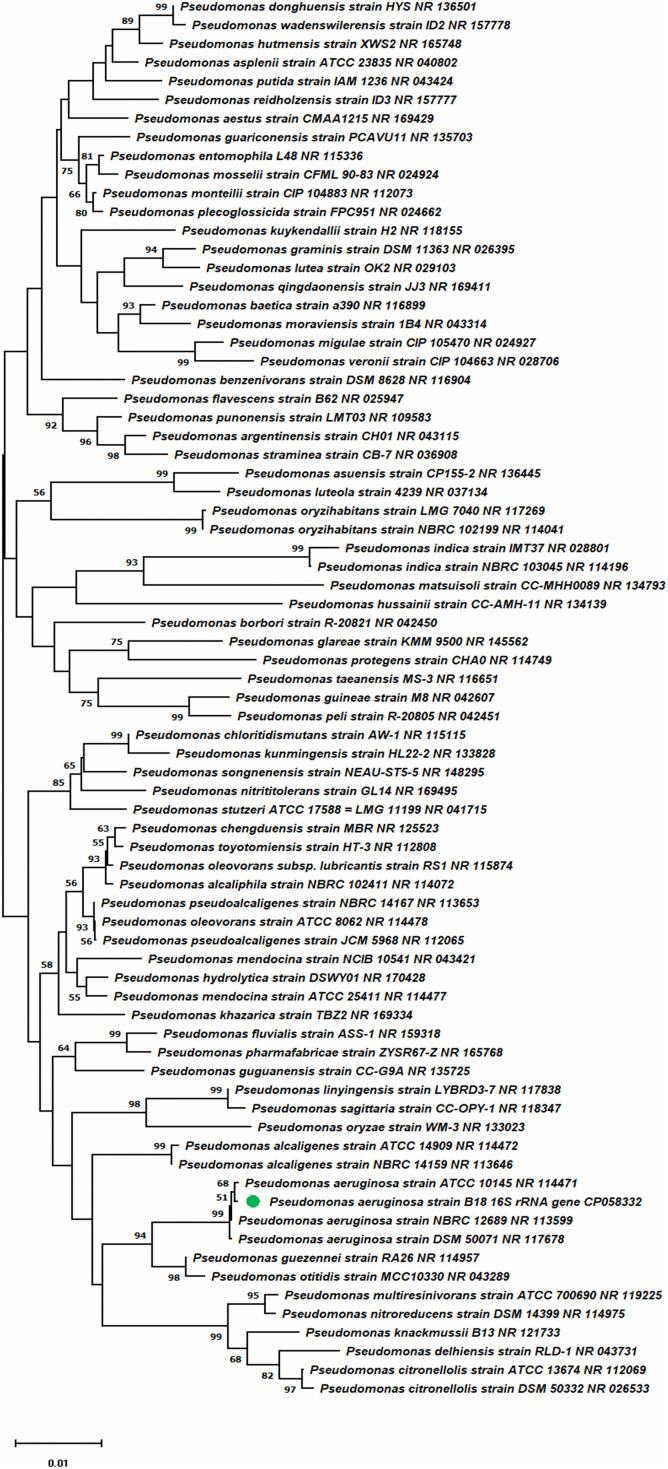
Phylogenetic tree presenting strain B18 position compared to other strains inside the genus *Pseudomonas*.

The genome of *P. aeruginosa* B18 included a circular chromosome of 6,490,014 base pairs with an average G + C content of 66.33% (Figure 4). The total predicted genes include approximately 5919 protein-coding genes (CDS), 65 tRNAs, and 12 rRNA genes. Furthermore, the characterization of predicted genes against different databases, *i.e*., COG, KEGG, GO, reference sequences (Refseq), and Pfam were 4512, 3245, 3601, 5882, and 5172 (Supplementary Figures 1–3 and Table 2). Clustered, regularly interspaced short palindromic repeats (CRISPR) are parts of prokaryotic DNA containing short base sequence repetitions and CRISPR-related genes (Cas gene) form a CRISPR-Cas system, which is an important defense system for organisms against foreign invaders. A total of three CRISPRs were also predicted from the B18 genome with 3,426 bp length (Figure 4 and Table 2). The assembled and annotated strain B18 genome sequence information was deposited in GenBank with accession number CP058332. Phylogenetic analysis of strain B18 showed its similarity 99% with *P. aeruginosa* strains (ATCC-10145, NBRC-12789, and DSM-50071) and placed the isolate in the *P. aeruginosa* clade and hence confirmed its identity as *P. aeruginosa* (Figure 5).

**TABLE 2:** Genome properties of *Pseudomonas aeruginosa* B18.

Features	Value
Genome size (bp)	6,490,014
GC content (%)	66.33
Topology	Circular
tRNA	65
rRNA (5S, 16S, 23S)	4, 4, 4
Protein-coding genes (CDS)	5919
Genes allocated to COG	4512
Genes allocated to GO	3601
Genes allocated to KEGG	3245
Genes allocated to Refseq	5882
Genes allocated to Pfam	5172
CRISPR	3

### Plant Growth-Promoting and Biocontrol-Related Genes in *P. aeruginosa* B18 Genome

**FIGURE 6:**
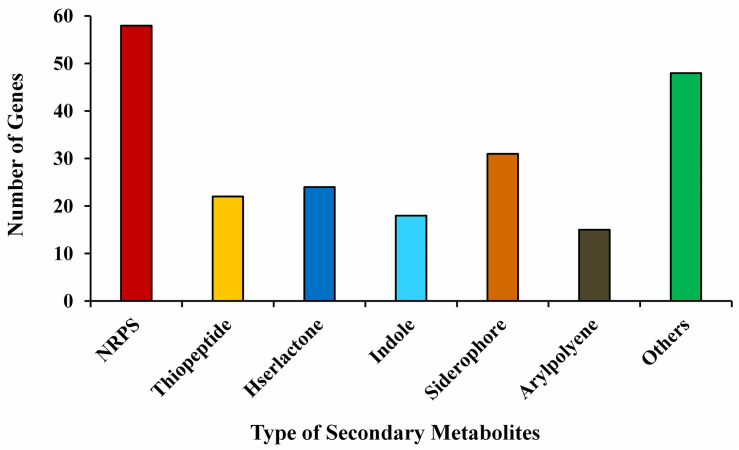
Secondary metabolites gene clusters in the genome of *Pseudomonas aeruginosa* B18.

The annotation of the *P. aeruginosa* B18 genome identified several genes related to ACC deaminase activity, nitrogen (*nifU*, *norBCDERQ*, *gltBDPS*, and *aatJMPQ*) and phosphate (*TC.PIT*, *pstABCS*, and *phoBDHRU*) metabolism, and production of IAA (*trpABCDEG*) and siderophores (*fes*, *entD*, and *fepA*). Additionally, there were genes related to methanethiol (*metH*), isoprene (*gcpE* and *ispE*), hydrolase (*ribA*, *folE2*, *gdhA*, and *bglBX*, *malO*), oxidoreductase (*SODA*, *osmC*, and *katE*), hydrogen cyanide (*hcnABC*), phenazine (*phzA_B* and *phzDFGMS*), salicylate (*pchA*), chitinase activity (*nagA*), exopolysaccharides (*algABDEFGIKLX* and *agl44*), metabolism (*acoABR* and *ACO*), 2,3-butanediol (*ilyABCDEG*), colonization (*minCDE, lysC*, and *yjbB*), and biofilm formation (*efp, hfq, flgBCDEFGHI*, and *motAB*) also present in the genome of B18, which might be involved in plant growth enhancement and biocontrol mechanisms (Table 3 and Supplementary Figure 4). PCR amplification results also confirmed that strain was positive for *nifH*, *acdS*, *hcn*, *prn*, and *phCA* genes with an approximate band size of 360, 755, 587, 786, and 1150 bp, respectively. All nucleotide sequences were submitted to the NCBI GenBank database with accession numbers MW027642 (*nifH*), MW027643 (*acdS*), MW027644 (*hcn*), and MW027645 (*prn*) except *phCA*. The secondary-metabolite gene clusters (antiSMASH) study showed the presence of several predicted gene clusters, *i.e*., NRPS, thiopeptide, hserlactone, indole, siderophore, aryl-polyene, and others in the B18 genome, as displayed in Figure 6.

**TABLE 3:** Genes linked to plant growth promotion and biocontrol activities in the endophyte B18 genome.

PGP Activities	Gene name	Gene Annotation	E. C. Number	Chromosome Location
**ACC Deaminase**	−	1-aminocyclopropane-1-carboxylate deaminase	3.5.99.7	6166698-6167597 −
**Nitrogen Metabolism**				
Nitrogen Fixation	*nifU*	Nitrogen fixation protein NifU and related proteins	−	466486-466872−
	*norD*	Nitric oxide reductase NorD protein	−	1131515-1133353 −
	*norB*	Nitric oxide reductase subunit B	1.7.2.5	1133355-1134752−
Ammonia Assimilation	*gltB*	Glutamate synthase (NADPH/NADH) large chain	1.4.1.13 1.4.1.14	2307717-2312162 +
	*gltD*	Glutamate synthase (NADPH/NADH) small chain	1.4.1.13 1.4.1.14	2312191-2313624 +
	*gltS*	Glutamate:Na + symporter, ESS family	−	6255382-6256596 +
	*gltP, gltT*	Proton glutamate symport protein	−	1806593-1807927 +
**Siderophores**				
Siderophore Enterobactin	*fes*	Enterochelin esterase and related enzymes	−	5314316-5315896−
	*entD*	Enterobactin synthetase component D	6.3.2.14	3777575-3778303−
	*fepA*	Ferric enterobactin receptor	−	3538571-3540799 +
**Plant Hormones**				
IAA Production	*trpC*	Indole-3-glycerol phosphate synthase	4.1.1.48	1025100-1025936−
	*trpD*	Anthranilate phosphor ribosyltransferase	2.4.2.18	1025933-1026982−
	*trpE*	Anthranilate synthase component I	4.1.3.27	1044724-1046202−
	*trpB*	Tryptophan synthase beta chain	4.2.1.20	1674493-1675701 +
	*trpA*	Tryptophan synthase alpha chain	4.2.1.20	1675698-1676504 +
	*trpE*	Anthranilate synthase component I	4.1.3.27	3595338-3596909 +
**Phosphate Metabolism**				
	*TC.PIT*	Inorganic phosphate transporter, PiT family	−	2115089-2116357 +
	*pstS*	Phosphate transport system substrate-binding protein	−	5448298-5449683 +
	*pstA*	Phosphate transporter permease subunit PtsA	−	1942356-1944032 +
	*pstB*	Phosphate ABC transporter ATP-binding protein	3.6.3.27	1944048-1944881 +
	*phoU*	Phosphate-specific transport system accessory protein PhoU	−	1944977-1945705 +
	*phoD*	Alkaline phosphatase D	−	570477-572039−
	*phoB*	Two-component system, OmpR family, phosphate regulon response regulator PhoB	−	1950623-1951312−
	*phoR*	Two-component system, OmpR family, phosphate regulon sensor histidine kinase PhoR	2.7.13.3	1949219-1950550−
	*phoH*	Phosphate starvation-inducible protein PhoH and related proteins	−	652180-653202 +
**Hydrolase**				
	*ribA*	GTP cyclohydrolase II	3.5.4.25	740436-741053−
	*folE2*	GTP cyclohydrolase I FolE	3.5.4.16	1745427-1746323−
	*gdhA*	Glutamate dehydrogenase (NADP +)	1.4.1.4	2851084-2852421 +
	*bglB*	Beta-glucosidase	3.2.1.21	2446884-2448425−
	*bglX*	Beta-glucosidase	3.2.1.21	4404697-4406991−
	*malQ*	4-alpha-glucanotransferase	2.4.1.25	4915439-4917493−
	*treS*	Maltose alpha-D-glucosyltransferase/alpha-amylase	5.4.99.16 3.2.1.1	48990024-4902304 +
**Biofilm Formation**				
	*efp*	Elongation factor P	−	5903161-5903727−
	*flgB*	Flagellar biosynthesis protein FlgB	−	3675639-3676046 +
	*flgC*	Flagellar basal body rod protein FlgC	−	3676052-3676492 +
	*flgD*	Flagellar basal body rod modification protein	−	3676505-3677218 +
	*flgE*	Flagellar hook protein FlgE	−	3677246-3678634 +
	*flgF*	Flagellar basal body rod protein FlgF	−	3678852-3679601 +
	*flgG*	Flagellar basal body rod protein FlgG	−	3679648-3680433 +
	*flgH*	Flagellar basal body L-ring protein	−	3680479-3681174 +
	*flgI*	Flagellar basal body P-ring protein	−	3681186-3682295 +
	*motA*	Chemotaxis protein MotA	−	2419987-2420838 +
	*motB*	Chemotaxis protein MotB	−	2420858-2421901 +
	*hfq*	RNA-binding protein Hfq	−	2431214-2431462 +
**Biocontrol**				
Hydrogen Cyanide	*hcnA*	Hydrogen cyanide synthase HcnA	1.4.99.5	4938725-4939039 +
	*hcnB*	Hydrogen cyanide synthase HcnB	1.4.99.5	4939036-4940430 +
	*hcnC*	Hydrogen cyanide synthase HcnC	1.4.99.5	4940433-4941686 +
	*phzM*	Phenazine-1-carboxylate N-methyltransferase	2.1.1.327	927364-928368−
	*phzA_B*	Phenazine biosynthesis protein	−	929583-930071 +
	*phzD*	Trans-2,3-dihydro-3-hydroxyanthranilic acid synthase	3.3.2.15	931321-931944 +
	*phzF*	Trans-2,3-dihydro-3-hydroxyanthranilate isomerase	5.3.3.17	933838-934677 +
	*phzG*	Di-hydrophenazine dicarboxylate synthase	1.10.3.16	934703-935347 +
	*phzS*	5-methylphenazine-1-carboxylate 1-monooxygenase	1.14.13.218	935527-936735 +
Salicylate	*pchA*	Salicylate biosynthesis isochorismate synthase	5.4.4.2	960348-961778 +
Chitinase Activity	*nagA*	N-acetylglucosamine-6-phosphate deacetylase	3.5.1.25	399348-400439 +
Exopolysaccharides	*algD*	GDP-mannose 6-dehydrogenase	1.1.1.132	149271-150581 +
	*alg44*	Mannuronan synthase	2.4.1.33	152288-153457 +
	*algK*	Alginate biosynthesis protein AlgK	−	153471-153471 +
	*algE*	Alginate production protein	−	154895-156367 +
	*algG*	Mannuronan 5-epimerase	5.1.3.37	156388-158019 +
	*algX*	Alginate biosynthesis protein AlgX		158032-159456 +
	*algL*	Poly(beta-D-mannuronate) lyase	4.2.2.3	159460-160563 +
	*algB*	Two-component system, NtrC family, response regulator AlgB	−	1803407-1804756−
	*algA*	Mannose-1-phosphate guanylyltransferase/mannose-6-phosphate isomerase	2.7.7.13 5.3.1.8	1834998-1836437 +
**Volatiles**				
Metabolism	*acoR*	Sigma-54 dependent transcriptional regulator, acetoin Dehydrogenase Operon transcriptional activator acor	−	849475-851352−
	*acoA*	Acetoin:2,6-dichlorophenolindophenol oxidoreductase subunit alpha	1.1.1.	853544-854518 +
	*acoB*	Acetoin:2,6-dichlorophenolindophenol oxidoreductase subunit beta	1.1.1.	854551-855570 +
	*acoR*	Sigma-54 dependent transcriptional regulator, acetoin dehydrogenase Operon transcriptional activator acor	−	689423-691354−
2,3-Butanediol	*ilvB*	Acetolactate synthase I/II/III large subunit	2.2.1.6	2714584-2716308 +
	*ilvH*	Acetolactate synthase I/III small subunit	2.2.1.6	2716311-2716802 +
	*ilvA*	Threonine dehydratase	4.3.1.19	1341468-1342982−
	*ilvC*	Ketol-acid reductoisomerase	1.1.1.86	2716845-2717861 +
	*ilvD*	Dihydroxy-acid dehydratase	4.2.1.9	1317186-1317186 +
	*ilvE*	Branched chain amino acid aminotransferase	2.6.1.42	2346991-2347914 +
Methanethiol	*metH*	5-methyltetrahydrofolate–homocysteine methyltransferase	2.1.1.13	4538474-4542178−
Isoprene	*ispG/gcpE*	4-hydroxy-3-methylbut-2-en-1-yl diphosphate synthase	1.17.7.1 1.17.7.3	458366-459481−
	*ispE*	4-(cytidine 5’-diphospho)-2-C-methyl-D-erythritol kinase	2.7.1.148	2752069-2752917−
**Colonization**				
	*lysC*	Aspartate kinase	2.7.2.4	3509930-3511168 +
	*minC*	Septum site-determining protein MinC	−	6320005-6320796−
	*minD*	Septum site-determining protein MinD	−	6320858-6321673 +
	*minE*	Cell division topological specificity factor	−	6321670-6321924 +
**Oxidoreductase**				
	*SODA*	Superoxide dismutase, Fe-Mn family	1.15.1.1	3012217-3012828 +
	*osmC*	Osmotically inducible protein osmc	−	1639449-1639874−
	*katE*	Catalase	1.11.1.6	2818107-2819648 +

### Comparative Genome Analysis

**FIGURE 7:**
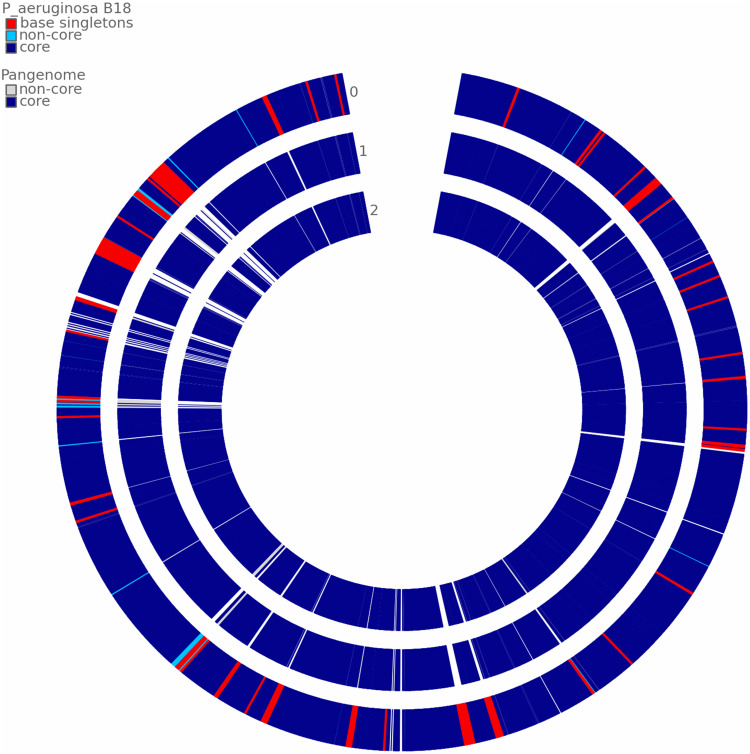
Comparative pangenome analysis of strain B18 against the reference genome *Pseudomonas aeruginosa* PA01 and *Pseudomonas* sp. PL10. The analysis shows differentiated genes in the core genome and non-core in blue and white colors, respectively. 0 = *P. aeruginosa* B18, 1 = *P. aeruginosa* PA01 and 2 = *Pseudomonas* sp. PL10.

According to ANI values, the B18 genome represents 99.18% and 98.74% similarity to *P. aeruginosa* PA01 and *Pseudomonas* sp. PL10, respectively, confirmed that B18 belongs to *P. aeruginosa* (Supplementary Figure 5). Pangenome analysis showed that all three genomes are closely related and highly similar (Figure 7). Pangenome analysis for base genome (*P. aeruginosa* PA01 and *Pseudomonas* sp. PL10) differentiated genes in core genome and non-core in blue and white colors, respectively. Comparative pangenome analysis with strain B18 genome showed that singleton genes (number of genes with no sequence homology to genes in any other genomes) were distributed all over the genome, but the core genome is highly similar. The analysis resulted in a total of 17477 protein-coding genes, include 16767 genes from homologs and 710 singletons. All the genes belong to 6148 families, which include 5438 homolog families and 710 singleton families. Results showed that strain *P. aeruginosa* B18 and *Pseudomonas* spp. PL10 was comparatively similar in gene composition that *P. aeruginosa* PA01 (Table 4).

**TABLE 4:** PanGenome genome features and comparative analysis of *Pseudomonas aeruginosa* B18 with reference *Pseudomonas* strains (*P. aeruginosa* PA01 and *Pseudomonas* spp. PL10).

Description	*P. aeruginosa* B18	*P. aeruginosa* PA01	*Pseudomonas* sp. PL10
Accession No	CP058332	AE004091	CP019338
Genome Size	6490014	6264404	6661962
Contig No	1	1	1
GC%	66.33%	66.55%	66.12%
Genes*	5981	5678	6076
CDS*	5950	5572	6057
Pseudogene*	49	19	60
tRNA*	64	63	62
rRNA*	12	13	12
tmRNA*	1	1	1
Genes	5901	5572	5997
Genes in Homologs	5655	5467	5635
Genes in Singletons	246	105	362
Homolog Families	5357	5204	5320

*Annotation is given as per Refseq and NCBI Microbial Genome annotation Pipeline.

### Greenhouse Assay of *P. aeruginosa* B18

**FIGURE 8:**
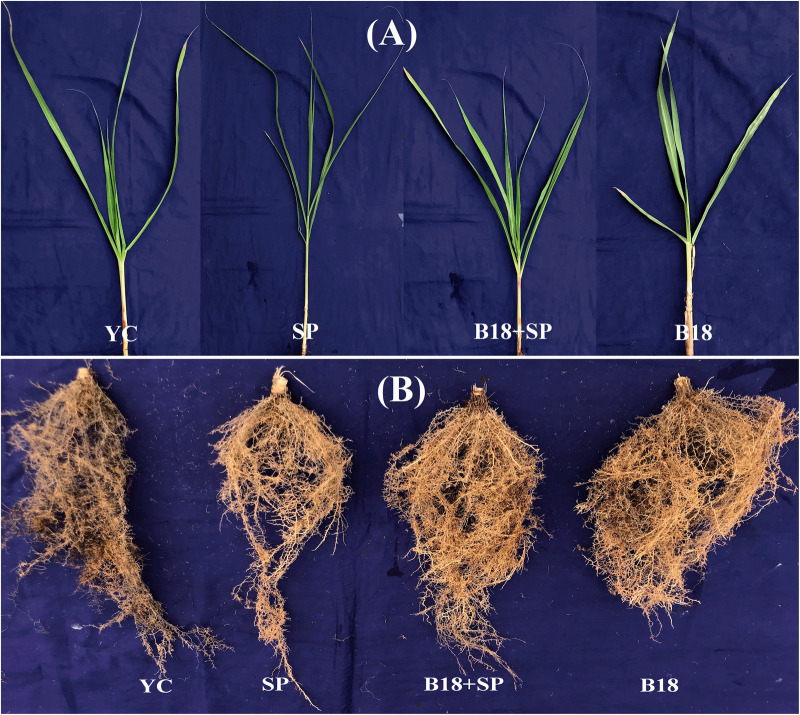
Plant growth promotion and biocontrol assay of Yacheng71-374 sugarcane variety 4 weeks after treatments **(A)** Overall shoot development and **(B)** Overall root development. YC (Yacheng71-374 sugarcane variety inoculated with sterile water), B18 (Yacheng71-374 sugarcane variety inoculated with strain B18), SP (Yacheng71-374 sugarcane variety inoculated with smut pathogen), and B18 + SP (Yacheng71-374 sugarcane variety inoculated with both B18 and smut pathogen).

The positive effect of endophytic strain B18 on sugarcane growth under smut pathogen stress conditions was evaluated in greenhouse assay. Plant height, shoot weight, root weight, chlorophyll content, leaf area, photosynthesis, transpiration rate, and stomatal conductance of sugarcane seedlings were measured 4 weeks after transplanting. The application of strain B18 (treatment B18) showed a positive increase in all growth parameters compared to all treatments (Figures 8A,B). The B18 strain, inoculated along with smut pathogen (treatment B18 + SP), decreased the impact of smut pathogen on the sugarcane plant, and all growth parameters were increased as compared to treatment SP (inoculated with smut pathogen) (Table 5). Moreover, a black whip, a typical response of sugarcane to smut, was observed in *S. scitamineum* inoculated sugarcane plants (treatment SP) after 3 months whereas plants in B18 + SP treatment showed no visual disease symptoms. These results proved that endophyte B18 could be used to control sugarcane smut as a plant growth promoter under smut stress conditions.

**TABLE 5:** Effect on plant growth parameters of Yacheng71-374 sugarcane variety with four treatments under greenhouse plant growth promotion and biocontrol assay.

Treatment	Chlorophyll	Leaf area	Height (cm)	Root weight (g)	Shoot weight (g)	Photosynthesis (μmoL CO_2_ m^–2^ s^–1^)	Transpiration rate (mmoL H_2_O m^–2^ s^–1^)	Stomatal conductance (mmoL H_2_O m^–2^ s^–1^)
YC	26.53 ± 0.39^c^	578.86 ± 8.56^c^	23.40 ± 0.35^c^	7.37 ± 0.11^c^	19.42 ± 0.29^c^	16.18 ± 0.24^c^	1.04 ± 0.02^c^	43.81 ± 0.65^c^
B18	41.38 ± 0.61^a^	818.47 ± 12.10^a^	28.64 ± 0.42^a^	10.93 ± 0.16^a^	27.94 ± 0.41^a^	28.44 ± 0.42^a^	2.87 ± 0.04^a^	86.53 ± 1.28^a^
SP	22.51 ± 0.33^d^	512.30 ± 7.58^d^	22.53 ± 0.33^d^	5.75 ± 0.09^d^	18.53 ± 0.27^d^	15.35 ± 0.23^d^	0.89 ± 0.01^d^	35.59 ± 0.53^d^
B18 + SP	28.46 ± 0.42^b^	753.28 ± 11.14^b^	24.82 ± 0.37^b^	7.90 ± 0.12^b^	20.31 ± 0.30^b^	22.43 ± 0.33^b^	1.13 ± 0.02^b^	53.65 ± 0.79^b^

### Effect on Phytohormones, Defense-Related Enzymes Activities, and Gene Expression

**FIGURE 9:**
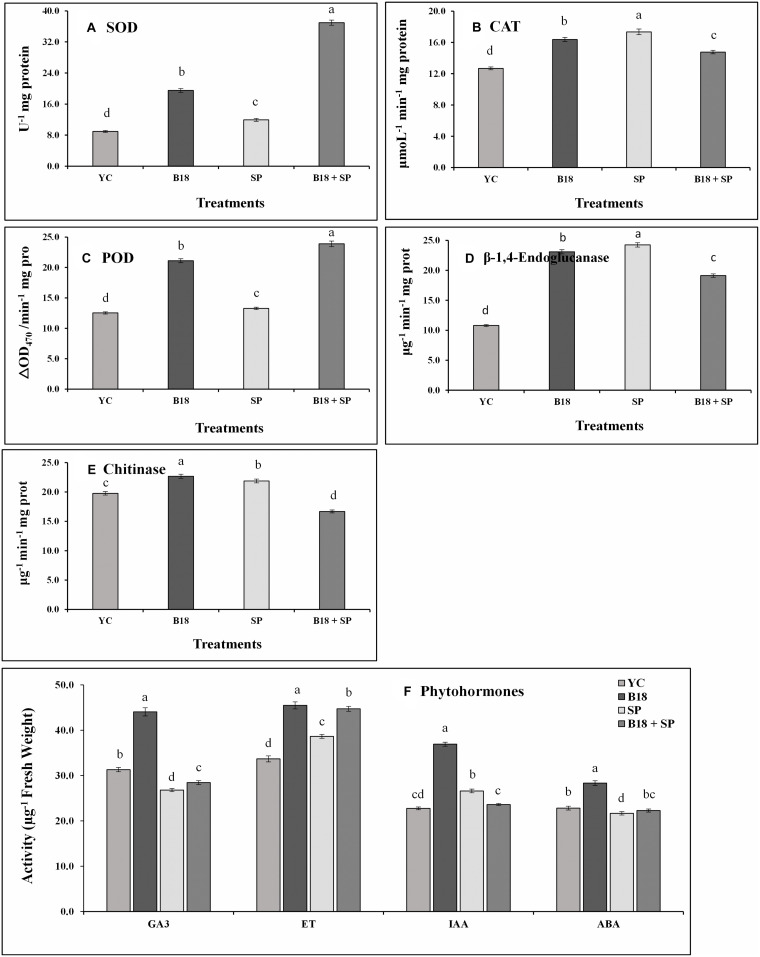
Effect on phytohormones and pathogen defense-related enzymes activities of Yacheng71-374 sugarcane variety with four treatments under greenhouse plant growth promotion and biocontrol assay **(A)** Superoxide dismutase, **(B)** Catalase, **(C)** Peroxidase, **(D)** β-1,4-Endoglucanase, **(E)** Chitinase, and **(F)** Phytohormones. The sum of all data points is described as the mean ± SE (*n* = 3). Dissimilar letters show significant differences between treatments (*p*-value < 0.05). YC (Yacheng71-374 sugarcane variety inoculated with sterile water), B18 (Yacheng71-374 sugarcane variety inoculated with strain B18), SP (Yacheng71-374 sugarcane variety inoculated with smut pathogen), and B18 + SP (Yacheng71-374 sugarcane variety inoculated with both B18 and smut pathogen).

We quantified the levels of GA_3_, ETH, ABA, and IAA, and SOD, CAT, POD, β-1,4-endoglucanase, and chitinase on the Yacheng71-374 sugarcane variety 4 weeks after treatment (Figure 9). *S. scitamineum* interaction increased the harmful ROS impact in sugarcane plant cells, and, to evade injurious intracellular ROS concentrations, plants trigger the production of antioxidant enzymes SOD, CAT, and POD (Figures 9A–C). In this study, a similar pattern of POD and SOD enzyme activities were observed and increased in treatment B18 + SP followed by B18, SP, and YC treatments (Figures 9A,C). In addition, maximum CAT activity was observed in treatment SP and minimum in treatment YC (Figure 9B). β-1,4-endoglucanase and chitinase enzyme activities were also measured, and treatment SP showed the greatest while treatment YC showed the lowest β-1,4-endoglucanase activity (Figure 9D). For chitinase, treatments B18 and B18 + SP confirmed the highest and lowest activity (Figure 9E). All measured phytohormone content was significantly higher in treatment B18 when compared to other treatments (Figure 9F). In the case of the other three treatments, treatment YC showed more GA_3_ and ABA contents followed by treatments B18 + SP and SP (Figure 9F); the ET value was elevated in treatment B18 + SP after that SP and YC; and the IAA value was higher in treatment SP compared to treatments B18 + SP and YC (Figure 9F).

**FIGURE 10:**
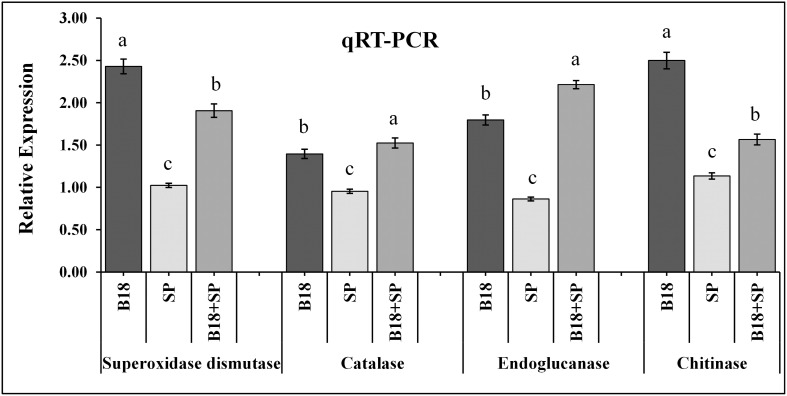
qRT-PCR expression analysis of pathogen defense-related genes in the leaf of smut susceptible sugarcane variety (Yacheng71-374) four weeks following treatments. The data were standardized to the level of the GAPDH expression. The mean ± SE is viewed as all data points (*n* = 3) and the same letters show no difference between treatments at *p*-value < 0.05.

The relative expression pattern of the *SuCHI, SuGLU, SuSOD*, and *SuCAT* genes in leaf tissues of Yacheng71-374 sugarcane variety was analyzed by qRT-PCR at 4 weeks after inoculation (Figure 10). Results demonstrated an enhanced expression of all genes in Yacheng71-374 inoculated with both B18 and smut pathogen (treatment B18 + SP) compared to Yacheng71-374 inoculated with only smut pathogen (treatment SP). Maximum enhanced expression of *SuSOD* and *SuCHI* genes was found in treatment B18, whereas, *SuCAT* and *SuGLU* genes expression was observed to be highest in treatment B18 + SP.

## Discussion

Microorganisms living in the endo-rhizosphere and their role in plant growth enhancement under biotic stress management is gaining importance. The biocontrol method has been studied for several years in modern agriculture, and many beneficial microbes have been suggested for crop disease control. However, many biocontrol agents are selective to host species, type of pathogen, environmental conditions, soil types, seasons, etc. (Prasad et al., 2015). Therefore, to discover new biocontrol agents that can respond to a wide range of surroundings, a systematic analysis is required (Chandra et al., 2020). In this study, we isolated one root-associated endophytic bacteria *P. aeruginosa* B18, which survived under various stress conditions and exhibited PGP and biocontrol activities against sugarcane pathogens in both laboratory and greenhouse conditions. Further comprehensive genome analysis of *P. aeruginosa* B18 has opened up several prospects to the understanding of the mechanisms used by this bacterium to promote sugarcane growth and alleviate biotic (smut pathogenesis) stress. Earlier, many strains of *P. aeruginosa*, were showed to prevent an extensive range of phytopathogens and growth enhancement in several crops (Devi et al., 2017; Li et al., 2017; Chenniappan et al., 2019; Patel et al., 2019; Chandra et al., 2020). Plant growth-promoting endophytic bacteria utilize diverse ways to enter plant tissues; the roots represent the most general way of entrance of endophytic bacteria into their host plant (Passari et al., 2015). Previous studies also revealed that the effective colonization of the bacterial strains and *P. aeruginosa* Z5 play a significant role to prevent plant pathogens and growth improvement (Compant et al., 2010; Yasmin et al., 2014). The finding of root colonization by GFP-tagged endophyte *P. aeruginosa* B18, along with the existence of genes facilitating this process (*minCDE*, *lysC*, and *yjbB*) in its genome, supports its role in biological control and growth enhancement in sugarcane.

Endophytic bacterial strains support plant growth *via* diverse direct and indirect systems, such as producing IAA, cytokinin, and GA_3_ phytohormones (Hardoim et al., 2015), P- solubilization, siderophores secretion, and plant resistance to biotic and abiotic stresses (Rosenblueth and Martínez-Romero, 2006; Gaiero et al., 2013; Lebeis, 2014). In this study, strain B18 showed IAA production, and comprehensive genome analysis established the presence of *trpABCDEG* genes related to IAA production. The occurrence of tryptophan-linked genes in the genome of bacteria is well-established, and it is associated with IAA biosynthesis (Tadra-Sfeir et al., 2011; Gupta et al., 2014). Similar to our results, complete genome analysis of *Sphingomonas* sp. LK11 and *Enterobacter roggenkampii* ED5 showed the existence of *trpABD* and *trpBE* genes, responsible for IAA production (Asaf et al., 2018; Guo et al., 2020). Previously, *P. aeruginosa* strain NJ-15 has been reported to produced IAA and biocontrol activity (Bano and Musarrat, 2003). In another study, *P. aeruginosa* BG was shown to produce IAA and promote growth enhancement in Chickpea (Goswami et al., 2013). Consistent with these findings, in this study, we observed improved growth of sugarcane plant after B18.

Siderophore production and P- solubilization are important PGP traits, siderophores are organic compounds produced by microbes and plants under iron-deficit condition by chelating iron from the surroundings, which can be taken up by microbial and plant cells (Ahmed and Holmström, 2014; Hardoim et al., 2015). Siderophores improve iron acquisition and inhibit plant pathogens through iron competition (Zhang et al., 2020). Strain B18 was able to produce siderophores, and the genome encoded *fes*, *fepA*, and *entD* siderophore enterobactin genes, which suggests that the strain is directly linked to siderophore production. Kang et al. (2020) identified *fes*, *entFS*, and *fepBCDG* genes in the genome of *P. psychrotolerans CS51* strain, and Gupta et al. (2014) identified *pvd*, *fpvA*, *mbtH*, *acrAB*, and *fhu* genes in the genome of *P. putida* involved in siderophore production. Secondary metabolites gene clusters were also predicted in the B18 genome, which can indicate its capacity to limit the growth of phytopathogenic microorganisms by restraining the bioavailability of iron (Singh et al., 2011). In the same way, phosphate-solubilizing microbes can solubilize immobile phosphorus in soil and which is accessible for plants (Passari et al., 2015; Joe et al., 2016). Numerous strains of *Pseudomonas*, such as *P. aeruginosa*, *P. fluorescens*, *P. koreensis*, *P. entomophila*, and *P. brassicacearum*, have shown phosphate solubilizing abilities (Durairaj et al., 2017; Li et al., 2017; Chenniappan et al., 2019; Nelkner et al., 2019; Chandra et al., 2020). Phosphate specific transport (*pst*) is utilized for free inorganic phosphate transport in *P. putida*, *B. subtilis*, *Escherichia coli*, *Sphingomonas* sp. LK1, and *E. roggenkampii* ED5, and these were made of *pstABCS* genes with a two-component signal transduction system including *phoP*/*phoR* for phosphate uptake (Gupta et al., 2014; Xie et al., 2016; Asaf et al., 2018; Guo et al., 2020). In the present study, strain B18 genomic sequence analysis also illustrated the existence of *pstABCS* and *phoBDHRU* genes.

Some endophytic bacteria carry genes essential for biological nitrogen fixation (BNF) to convert dinitrogen gas (N_2_) into ammonium and nitrate inside the host plant (Bhattacharjee et al., 2008). Earlier, many genera of bacterial genera isolated from sugarcane *P. aeruginosa, P. koreensis*, *P. entomophila, E. roggenkampii, Bacillus megaterium*, *B. mycoides*, *Kosakonia radicincitans*, and *Stenotrophomonas maltophilia* have enhanced the growth of their host plant in nutrient-deprived conditions (Li et al., 2017; Guo et al., 2020; Singh et al., 2020a, b). Similarly, strain B18 exhibited nitrogenase activity (11.38 ± 0.17 nmoL C_2_H_4_ mg protein h^–1^) with *nifH* amplification, and its genome comprises nitrogen metabolism-related genes, such as *nifU* (nitrogen fixation), *norBCDERQ* (nitrosative stress), and *gltBDPS*, and *aatJMPQ* (ammonia assimilation), confirming its nitrogen-fixing ability. Gene *nifU* is necessary for nitrogen fixation and takes part in the Fe-S cluster assembly (Smith et al., 2005). Similarly, Kang et al. (2020) detected *norB* gene in the genome of *P. psychrotolerans CS51*.

Endophytic bacteria protect host plants from various stresses by producing antimicrobial compounds and reducing the ethylene synthesis pathway by using ACC deaminase (Glick, 2014; Mercado-Blanco and Lugtenberg, 2014). This study confirmed that strain B18 demonstrated ACC deaminase activity as well as the presence of *acdS* gene at ∼755 bp. Similarly, Nelkner et al. (2019) and Gupta et al. (2014) reported a functional *acdS* gene in genome of *P. putida* and *P. brassicacearum* 3Re2-7 strains. Phenazine-1-carboxylic acid (*phCA*) and pyrrolnitrin (*prn*) are common antibiotics, while HCN is an antimicrobial compound produced by many bacterial strains involved in the inhibition of fungal plant pathogens. The qualitative test results showed the B18 strain had HCN and ammonia production ability as well as a confirmed occurrence of *hcn*, *phCA*, and *prn* antibiotic genes. In addition, the presence of HCN (*hcnABC*), phenazine (*phzA_B*, *phzDFGMS*, and salicylate *(pchA*) genes in its genome indicate that this bacterium could confer host plant resistance to pathogens. This result is similar to the findings of Chenniappan et al. (2019) for the production of pyrrolnitrin and volatile antifungal compound HCN in *P. aeruginosa* PGP strains. Nelkner et al. (2019) also confirmed gene clusters coding for hydrogen cyanide (*hcnABC*) in the genome of *P. brassicacearum* 3Re2-7, and Gupta et al. (2014) identified the *phzF* gene in *P. putida* genome involved in phenazine synthesis.

Microbial biofilms are surrounded by self-generating extracellular polymeric substances (EPSs) that allow microorganisms to adjust and survive under adverse conditions (Costerton et al., 1995). Biofilm development is the main characteristic that has been connected to the colonization capacity of biocontrol microbes (Ongena and Jacques, 2008). In this study, we searched the genes *efp*, *hfq*, *flgBCDEFGHI*, and *motAB* involved in biofilm formation in the genome of strain B18. Previous studies also reported the presence of biofilm-related genes in *Escherichia coli* K-12, *P. aeruginosa* PAO1, *P. polymyxa*, and *P. chlororaphis* subsp. *aurantiaca* JD37 strains (Domka et al., 2007; Hu et al., 2011; Luo et al., 2018; Zhang et al., 2020). Some bacteria, such as *B. cereus* (Xu et al., 2014), *Paenibacillus* (Timmusk et al., 2015), and *P. stutzeri* (Wang et al., 2017), exhibited biocontrol activity against phytopathogens by forming biofilm-like structures.

Biotic stress also affects plant yield by increasing the level of intracellular ROS, causing tissue damage, and the production and exclusion of ROS in plants are retained by the activity of several antioxidant enzymes (Gupta and Datta, 2003). Bacteria produce cell-wall-degrading enzymes and different metabolites that prevent the growth of pathogenic microbes (Shoda, 2000; Chernin and Chet, 2002). Certain bacteria activate a phenomenon identified as ISR to refer to stress-related physical and chemical adaptations in plants against pathogens attack. The metabolic pathways in plants are either up-or downregulated under stress at diverse developmental stages, altering plant growth (Chaves et al., 2002). The majority of information on ISR is associated with rhizobacterial strain, but some endophytic bacteria have also been reported to include ISR activity. For instance, *P. fluorescens* EP1 activated ISR in response to *Colletotrichum falcatum* pathogen causing sugarcane red rot disease (Viswanathan and Samiyappan, 1999).

Previously, a number of endophytic bacteria such as *Bacillus*, *Burkholderia*, *Enterobacter*, *Pseudomonas*, and *Serratia* have been considered to be effective in controlling various plant pathogens (Mercado-Blanco and Lugtenberg, 2014; Esmaeel et al., 2016; Larran et al., 2016; Kandel et al., 2017; Guo et al., 2020). However, little is known about the use of fungal and bacterial isolates for biocontrol of sugarcane smut disease (Liu et al., 2017). More significantly, symbiotic plants with these bacterial endophytes were not only able of mitigate the stress, it could also improve plant weight and height (Rojas-Tapias et al., 2012; Naveed et al., 2014; Yaish et al., 2015). However, the mechanisms by which endophytic bacteria alleviate biotic stress still remain unclear. Endophytic bacteria can offer numerous benefits to the host plant by producing phytohormones and pathogen defense-related enzymes. Hence to determine whether B18 could colonize sugarcane plants as endophytes and supports plant growth under pathogen stress, we performed a pot experiment under greenhouse conditions. Our results demonstrated a significant interactive effect of strain B18 and smut pathogen (SP) inoculation with IAA, GA3, ET, and ABA in smut susceptible sugarcane variety Yacheng71-374. Sugarcane seedlings inoculated with B18 significantly increased all phytohormone levels, whereas B18 + SP inoculation exhibited higher GA3, ET, and ABA values than that of SP inoculation. Decreased production of ABA was observed in plants during pathogen infection (Fan et al., 2009). Phytohormones CYT, ETH, and ABA play an essential part in the plant’s response to pathogen attack and environmental stresses (Sauter et al., 2001; Bari and Jones, 2009). These hormones support plant growth, improve root growth, enhance fertilizer and water uptake and assimilation, and participate in diverse metabolic activities related to abiotic and biotic stresses (Graham, 2003; Stepanova et al., 2007). The systemic resistance stimulated by microorganisms enhances plant health under multiple-stresses (Sathya et al., 2017). Accordingly, in this study, B18 inoculation in smut susceptible sugarcane seedlings had a positive effect on all growth parameters, suggesting that it has important PGP and biocontrol activity under *S. scitamineum* stress conditions. Chenniappan et al. (2019) reported *P. aeruginosa* MML2424 enhanced growth and reduced the disease occurrence in turmeric plants as compared to farmyard manure in field conditions. In addition, the *in vivo* pot experiments under *S. scitamineum* stress showed that the strain enhanced plant stress tolerance by regulating the contents of hydrolytic and antioxidant enzymes. Glucanase and chitinase enzymes are active in response to many fungal diseases and play a vital role in biological and chemical defenses in plants (Maximova et al., 2006; Su et al., 2013). Catalase (CAT), POD, and, SOD are key defensive enzymes in plants and contribute to plant defense (Scandalios, 1993). Peroxidase (POD) belongs to the pathogen-related protein, and its expression is directly connected to plant disease resistance (Hiraga et al., 2001). In this study, B18 considerably improved the activities and expression of CAT, POD, SOD, chitinase, and endoglucanase in the Yacheng71-374 variety under *S. scitamineum* stress conditions. We also identified several defense-related genes in the genome of B18 such as hydrolase (*ribA*, *floE2*, *gdhA*, *bglBX*, and *malQ*) and oxidoreductase (*SODA*, *osmC*, and *KatE*), indicating that this endophytic bacterium can enhance plant growth under biotic stress. Similarly, genes for chitinase, SOD (*sodBC*), POD (*osmC* and *oxyR*), and CAT production were observed in the *P. putida* genome (Gupta et al., 2014).

## Conclusion

This is the first report of experimental confirmations of endophytic *P. aeruginosa* B18 strain as a biocontrol and PGP bacterium isolated from sugarcane root. This bacterium exhibited various PGP and antifungal activities as well as improved sugarcane growth under smut pathogen stress in greenhouse assays. Additionally, analysis of the *P. aeruginosa* strain B18 genome suggests that it encodes numerous potential genes implicated in plant growth promotion and biocontrol mechanisms, providing additional insights into the biological role of B18 in relation to plant growth and biocontrol. At this stage, little is known about the regulatory mechanisms controlling these genes. Evaluation of B18 in field trials is required to assess its performance under field conditions in sugarcane growth promotion and protection against smut disease. If successful, the B18 strain may become a cost-effective and eco-friendly biofertilizer for sustainable sugarcane crop production.

## Data Availability Statement

The datasets presented in this study can be found in online repositories. The names of the repository/repositories and accession number(s) can be found in the article/Supplementary Material.

## Author Contributions

Y-RL, PS, and RKS planned the proposal and designed the experiments. PS, RKS, and D-JG performed experiments. AS, RNS, D-PL, MM, and X-PS participated in data analysis. PS and RKS wrote the original draft preparation. L-TY, Y-RL, and PL revised and finalized this article. All authors contributed to the article and approved the submitted version.

## Conflict of Interest

RNS was employed by company AgriGenome Labs Pvt, Ltd. The remaining authors declare that the research was conducted in the absence of any commercial or financial relationships that could be construed as a potential conflict of interest.

## Footnotes

1 www.majorbio.com
