# Supplementary material for: Whole Genome Analysis of Sugarcane Root-Associated Endophyte Pseudomonas aeruginosa B18—A Plant Growth-Promoting Bacterium With Antagonistic Potential Against Sporisorium scitamineum
Source: Front Microbiol. 2021 Feb 5;12:628376. doi: 10.3389/fmicb.2021.628376 (PMC7894208; doi:10.3389/fmicb.2021.628376)
Supplement: Supplementary file 1 [file Data_Sheet_1.docx]

Supplementary Material

**Table S1.** List of media used in this study.

| **Media Name** | **Compositions** | **Quantity (gm L^-1^)** |
| --- | --- | --- |
| **Ashby’s glucose agar** | Glucose | 20 |
|  | Dipotassium phosphate | 0.2 |
|  | Magnesium sulphate | 0.2 |
|  | Sodium chloride | 0.2 |
|  | Calcium carbonate | 5 |
|  | Potassium sulphate | 0.1 |
|  | Agar | 18 |
|  | Final pH | 7.4 |
| **Nutrient agar** | Peptone | 5 |
|  | Beef extract | 3 |
|  | Sodium chloride | 5 |
|  | Agar | 18 |
|  | pH | 7.2 |
| **Potato dextrose agar** | Potato | 200 |
|  | Dextrose | 20 |
|  | Agar | 18 |
| **Luria bertani broth** | Yeast extract | 5 |
|  | Peptone | 10 |
|  | NaCl | 10 |
| **Dworkin and Foster salts minimal medium** | Glucose | 2 |
|  | Gluconic acid | 2 |
|  | Citric acid | 2 |
|  | KH_2_PO_4_ | 4 |
|  | Na_2_HPO_4_ | 6 |
|  | MgSO_4_**.**7H_2_O | 0.2 |
|  | CaCl_2_ | 200 mg |
|  | FeSO_4_.7H_2_O | 200 mg |
|  | H_3_BO_3_ | 15 mg |
|  | ZnSO_4_.7H_2_O | 20 mg |
|  | Na_2_MoO_4_ | 10 mg |
|  | KI | 10 mg |
|  | NaBr | 10 mg |
|  | MnCl_2_ | 10 mg |
|  | COCl_2_ | 5 mg |
|  | CuCl_2_ | 5 mg |
|  | AlCl_3_ | 2 mg |
|  | NiSO_4_ | 2 mg |
| **YEPS Broth (gm L^-1^)** | Yeast extract | 10 |
|  | Peptone | 20 |
|  | Sucrose | 20 |

**Table S2.** List of primers used in this study.

| **Gene** | **Primer** | **Sequence (5**′ **-------→ 3′)** | **Product size (bp)** | **References** |
| --- | --- | --- | --- | --- |
| *nifH* | Pol**-**F  Pol**-**R | TGCGAYCC-SAARGCBGACTC  ATSGCCATCATYTCRCCGGA | 360 | Poly et al., 2001 |
| *phCA* | PhCA-F  PhCA**-**R | TTGCCAAGCCTCGCTCCAAC  CCGCGTTGTTCCTCGTTCAT | 1150 | Raaijmakers et al., 1997 |
| *prn* | Prn**-**F  Prn**-**R | GGGGCGGGCCGTGGTGATGGA  YCCCGCSGCCTGYCTGGTCTG | 786 | Souza and Raaijmakers 2003 |
| *hcn* | HCN**-**F  HCN**-**R | ACTGCCAGGGGCGGATGTGC  ACGATGTGCTCGGCGTAC | 587 | Ramette et al., 2003 |
| *acdS* | ACD-F  ACD-R | GCAACAAGACGCGCAAGYTNGARTAYN T  GTGCATCGACTTGCCCTCRWANACNGG RT | 755 | Li et al., 2011 |
| **qRT-PCR Primer** | | | | |
| *GAPDH* | GAPDH-F  GAPDH-R | CTCTGCCCCAAGCAAAGATG  TGTTGTGCAGCTAGCATTG | qRT-PCR | (Niu et al., 2015) |
| *SuChi* | ScChi-QF  ScChi-QR | ACGGCTACGGCGACAACA  GTCCGCTGACCAGATGAAGAG | qRT-PCR | (Su et al., 2014 |
| *SuGluD1* | D-QF  D-QR | TGCTACTTCTTATCCACCCTCTG  CGTTGACATAGAAAGGTGAGCC | qRT-PCR | (Su et al., 2013) |
| *SuCAT* | CAT-F  CAT-R | CTTGTCTGGAGCACATACACTTGGA  TTCTCCGCATAGACCTTGAACTTTG | qRT-PCR | (Chen, 2012) |
| *SuSOD* | SOD-F  SOD-R | TTTGTCCAAGAGGGAGATGG  CTTCTCCAGCGGTGACATTT | qRT-PCR | (Jain et al., 2015) |

Su, Y.; Xu, L.; Fu, Z.; Yang, Y.; Guo, J.; Wang, S.; and Que.Y. (2014) *ScChi*, encoding an acidic class III chitinase of sugarcane, confers positive responses to biotic and abiotic stresses in sugarcane. *IJMS*. 15, 2738–2760.

Chen, S.S. (2012) Cloning and Expression Analysis of ROS Metabolism Pathway Key Genes from Sugarcane. Master’s Thesis, Fujian Agriculture and Forestry University, Fuzhou, China.

Jain, R.; Chandra, A.; Venugopalan, V.K.; and Solomon, S. (2015). Physiological Changes and Expression of SOD and P5CS Genes in Response to Water Deficit in Sugarcane. *Sugar Tech* 17, 276- 282.

**Table S3.** *In-vitro* abiotic stress tolerance ability of B18 strain (O.D at 600 nm).

| **NaCl** | 7 (%) | 8 (%) | 9 (%) | 10 (%) | 11 (%) | 12 (%) |
| --- | --- | --- | --- | --- | --- | --- |
|  | 0.930±0.012 | 0.235±0.003 | 0.106±0.001 | 0.079±0.001 | 0.083±0.001 | 0.074+0.001 |
| **pH** | 5 | 6 | 7 | 8 | 9 | 10 |
|  | 0.645±0.008 | 0.571±0.007 | 0.98±0.012 | 0.568±0.007 | 0.594±0.007 | 0.066±0.001 |
| **Temperature** | 20 (℃) | 25 (℃) | 30 (℃) | 35 (℃) | 40 (℃) | 45 (℃) |
|  | 0.636±0.008 | 0.666±0.008 | 0.541±0.007 | 0.78±0.01 | 0.744±0.009 | 0.467±0.006 |

**Table S4.** List of carbon, nitrogen, osmolytes, and pH present in each well of GENIII, PM3B, PM9, and PM10 BIOLOG plates.

| **S. No.** | **Serial No.** | **Carbon** | **Nitrogen** | **Osmolyte** | **pH** |
| --- | --- | --- | --- | --- | --- |
| 1 | A1 | Negative Control | Negative Control | NaCl 1% | pH 3.5 |
| 2 | A2 | Dextrin | Ammonia | NaCl 2% | pH 4 |
| 3 | A3 | D-Maltose | Nitrite | NaCl 3% | pH 4.5 |
| 4 | A4 | D-Trehalose | Nitrate | NaCl 4% | pH 5 |
| 5 | A5 | D-Cellobiose | Urea | NaCl 5% | pH 5.5 |
| 6 | A6 | Gentiobiose | Biuret | NaCl 5.5% | pH 6 |
| 7 | A7 | Sucrose | L -Alanine | NaCl 6% | pH 7 |
| 8 | A8 | D-Turanose | L-Arginine | NaCl 6.5% | pH 8 |
| 9 | A9 | Stachyose | L-Asparagine | NaCl 7% | pH 8.5 |
| 10 | A10 | Positive Control | L - Aspartic Acid | NaCl 8% | pH 9 |
| 11 | A11 | pH 6 | L-Cysteine | NaCl 9% | pH 9.5 |
| 12 | A12 | pH 5 | L-Glutamic Acid | NaCl 10% | pH 10 |
| 13 | B1 | D-Raffinose | L-Glutamine | NaCl 6% | pH 4.5 |
| 14 | B2 | α-D-Lactose | Glycine | NaCl 6% + Betaine | pH 4.5 + L-Alanine |
| 15 | B3 | D-Melibiose | L-Histidine | NaCl 6% +N-N Dimethyl glycine | pH 4.5 + L-Arginine |
| 16 | B4 | β-Methyl-D-Glucoside | L-Isoleucine | NaCl 6% + Sarcosine | pH 4.5 + L- Asparagine |
| 17 | B5 | D-Salicin | L-Leucine | NaCl 6% + Dimethyl sulphonyl propionate | pH 4.5 + L- Aspartic Acid |
| 18 | B6 | N-Acetyl-D-Glucosamine | L-Lysine | NaCl 6% + MOPS | pH 4.5 + L- Glutamic Acid |
| 19 | B7 | N-Acetyl-β-DMannosamine | L-Methionine | NaCl 6% + Ectoine | pH 4.5 + L-Glutamine |
| 20 | B8 | N-Acetyl-D-Galactosamine | L- Phenylalanine | NaCl 6% + Choline | pH 4.5 + Glycine |
| 21 | B9 | N-AcetylNeuraminic Acid | L-Proline | NaCl 6% + Phosphoryl choline | pH 4.5 + L-Histidine |
| 22 | B10 | 1% NaCl | L-Serine | NaCl 6% + Creatine | pH 4.5 + L-Isoleucine |
| 23 | B11 | 4% NaCl | L-Threonine | NaCl 6% + Creatinine | pH 4.5 + L-Leucine |
| 24 | B12 | 8% NaCl | L-Tryptophan | NaCl 6% + L - Carnitine | pH 4.5 + L-Lysine |
| 25 | C1 | α-D-Glucose | L-Tyrosine | NaCl 6% + KCl | pH 4.5 + L-Methionine |
| 26 | C2 | D-Mannose | L -Valine | NaCl 6% + L-proline | pH 4.5 + L-Phenylalanine |
| 27 | C3 | D-Fructose | D-Alanine | NaCl 6% + N -Acethyl L-glutamine | pH 4.5 + L-Proline |
| 28 | C4 | D-Galactose | D-Asparagine | NaC1 6% + β-Glutamic acid | pH 4.5 + L-Serine |
| 29 | C5 | 3-Methyl Glucose | D-Aspartic Acid | NaC1 6% + γ –Amino -n -butyric acid | pH 4.5 + L-Threonine |
| 30 | C6 | D-Fucose | D-Glutamic Acid | NaC1 6% + Glutathione | pH 4.5 + L-Tryptophan |
| 31 | C7 | L-Fucose | D-Lysine | NaCl 6% + Glycerol | pH 4.5 + L-Tyrosine |
| 32 | C8 | L-Rhamnose | D-Serine | NaC1 6% + Trehalose | pH 4.5 + L-Valine |
| 33 | C9 | Inosine | D-Valine | NaC1 6% + Trimethylamine -N-oxide | pH 4.5 + Hydroxy- L-Proline |
| 34 | C10 | 1% Sodium Lactate | L-Citrulline | NaC1 6% + Trimethylamine | pH 4.5 + L-Ornithine |
| 35 | C11 | Fusidic Acid | L-Homoserine | NaCl 6% + Octopine | pH 4.5 + L-Homoarginine |
| 36 | C12 | D-Serine | L-Ornithine | NaC1 6% + Trigonelline | pH 4.5 + L-Homoserine |
| 37 | D1 | D-Sorbitol | N - Acetyl-D, L-Glutamic Acid | Potassium chloride 3% | pH 4.5 + Anthranilic acid |
| 38 | D2 | D-Mannitol | N-Phthaloyl-L Glutamic Acid | Potassium chloride 4% | pH 4.5 + L-Norleucine |
| 39 | D3 | D-Arabitol | L-Pyroglutamic Acid | Potassium chloride 5% | pH 4.5 + L-Norvaline |
| 40 | D4 | myo-Inositol | Hydroxylamine | Potassium chloride 6% | pH 4.5 + L-α- Amino - N- butyric acid |
| 41 | D5 | Glycerol | Methylamine | Sodium sulfate 2% | pH 4.5 + L- p - Aminobenzoate |
| 42 | D6 | D-Glucose-6-PO_4_ | N-Amylamine | Sodium sulfate 3% | pH 4.5 + L- Cysteic acid |
| 43 | D7 | D-Fructose-6-PO_4_ | N-Butylamine | Sodium sulfate 4% | pH 4.5 + D-Lysine |
| 44 | D8 | D-Aspartic Acid | Ethylamine | Sodium sulfate 5% | pH 4.5 + 5-Hydroxy Lysine |
| 45 | D9 | D-Serine | Ethanolamine | Ethylene glycol 5% | pH 4.5 + 5-Hydroxy Tryptophan |
| 46 | D10 | Troleandomycin | Ethylenediamine | Ethylene glycol 10% | pH 4.5 + D, L-Diamino pimelic acid |
| 47 | D11 | Rifamycin SV | Putrescine | Ethylene glycol 15% | pH 4.5 + Trimethyl amine-N-oxide |
| 48 | D12 | Minocycline | Agmatine | Ethylene glycol 20% | pH 4.5 + Urea |
| 49 | E1 | Gelatin | Histamine | Sodium formate 1% | pH 9.5 |
| 50 | E2 | Glycyl-L-Proline | β-Phenylethyl-amine | Sodium formate 2% | pH 9.5 + L-Alanine |
| 51 | E3 | L-Alanine | Tyramine | Sodium formate 3% | pH 9.5 + L-Arginine |
| 52 | E4 | L-Arginine | Acetamide | Sodium formate 4% | pH 9.5 + L-Asparagine |
| 53 | E5 | L-Aspartic Acid | Formamide | Sodium formate 5% | pH 9.5 + L-Aspartic Acid |
| 54 | E6 | L-Glutamic Acid | Glucuronamide | Sodium formate 6% | pH 9.5 + L-Glutamic Acid |
| 55 | E7 | L-Histidine | D, L-Lactamide | Urea 2% | pH 9.5 + L-Glutamine |
| 56 | E8 | L-Pyroglutamic Acid | D-Glucosamine | Urea 3% | pH 9.5 + Glycine |
| 57 | E9 | L-Serine | D-Galactosamine | Urea 4% | pH 9.5 + L-Histidine |
| 58 | E10 | Lincomycin | D-Mannosamine | Urea 5% | pH 9.5 + L-Isoleucine |
| 59 | E11 | Guanidine HCl | N-Acetyl-D-Glucosamine | Urea 6% | pH 9.5 + L-Leucine |
| 60 | E12 | Niaproof 4 | N-Acetyl-D-Galactosamine | Urea 7% | pH 9.5 + L-Lysine |
| 61 | F1 | Pectin | N-Acetyl-D-Mannosamine | Sodium Lactate 1% | pH 9.5 + L-Methionine |
| 62 | F2 | D-Galacturonic Acid | Adenine | Sodium Lactate 2% | pH 9.5 + L-Phenylalanine |
| 63 | F3 | L-Galactonic Acid Lactone | Adenosine | Sodium Lactate 3% | pH 9.5 + L-Proline |
| 64 | F4 | D-Gluconic Acid | Cytidine | Sodium Lactate 4% | pH 9.5 + L-Serine |
| 65 | F5 | D-Glucuronic Acid | Cytosine | Sodium Lactate 5% | pH 9.5 + L-Threonine |
| 66 | F6 | Glucuronamide | Guanine | Sodium Lactate 6% | pH 9.5 + L-Tryptophan |
| 67 | F7 | Mucic Acid | Guanosine | Sodium Lactate 7% | pH 9.5 + L-Tyrosine |
| 68 | F8 | Quinic Acid | Thymine | Sodium Lactate 8% | pH 9.5 + L-Valine |
| 69 | F9 | D-Saccharic Acid | Thymidine | Sodium Lactate 9% | pH 9.5 + Hydroxy- L-Proline |
| 70 | F10 | Vancomycin | Uracil | Sodium Lactate 10% | pH 9.5 + L-Ornithine |
| 71 | F11 | Tetrazolium Violet | Uridine | Sodium Lactate 11% | pH 9.5 + L-Homoarginine |
| 72 | F12 | Tetrazolium Blue | Inosine | Sodium Lactate 12% | pH 9.5 + L-Homoserine |
| 73 | G1 | p-Hydroxy- Phenylacetic Acid | Xanthine | Sodium Phosphate pH 7 20mM | pH 9.5 + Anthranilic acid |
| 74 | G2 | Methyl Pyruvate | Xanthosine | Sodium Phosphate pH 7 50mM | pH 9.5 + L-Norleucine |
| 75 | G3 | D-Lactic Acid Methyl Ester | Uric Acid | Sodium Phosphate pH 7 100mM | pH 9.5 + L-Norvaline |
| 76 | G4 | L-Lactic Acid | Alloxan | Sodium Phosphate pH 7 200mM | pH 9.5 + Agmatine |
| 77 | G5 | Citric Acid | Allantoin | Sodium Benzoate pH 5.2 20mM | pH 9.5 + Cadaverine |
| 78 | G6 | α-Keto-Glutaric Acid | Parabanic Acid | Sodium Benzoate pH 5.2 50mM | pH 9.5 + Putrescine |
| 79 | G7 | D-Malic Acid | D, L-α-Amino-N-Butyric Acid | Sodium Benzoate pH 5.2 100mM | pH 9.5 + Histamine |
| 80 | G8 | L-Malic Acid | γ-Amino-N-Butyric Acid | Sodium Benzoate pH 5.2 200mM | pH 9.5 + Phenylethylamine |
| 81 | G9 | Bromo-Succinic Acid | ε-Amino-N-Caproic Acid | Ammonium sulfate pH8 10mM | pH 9.5 + Tyramine |
| 82 | G10 | Nalidixic Acid | D, L-α-Amino-Caprylic Acid | Ammonium sulfate pH8 20mM | pH 9.5 + Creatine |
| 83 | G11 | Lithium Chloride | δ-Amino-N-Valeric Acid | Ammonium sulfate pH8 50mM | pH 9.5 + Trimethyl amine- N-oxide |
| 84 | G12 | Potassium Tellurite | α-Amino-N-Valeric Acid | Ammonium sulfate pH8 100mM | pH 9.5 + Urea |
| 85 | H1 | Tween 40 | Ala-Asp | Sodium Nitrate 10mM | X-Caprylate |
| 86 | H2 | γ-Amino-ButryricAcid | Ala-Gln | Sodium Nitrate 20mM | X–α-D-Glucoside |
| 87 | H3 | α-Hydroxy- Butyric Acid | Ala-Glu | Sodium Nitrate 40mM | X-β-D-Glucoside |
| 88 | H4 | β-Hydroxy-D,LButyricAcid | Ala-Gly | Sodium Nitrate 60mM | X-α-D-Galactoside |
| 89 | H5 | α-Keto-Butyric Acid | Ala-His | Sodium Nitrate 80mM | X-β-D-Galactoside |
| 90 | H6 | Acetoacetic Acid | Ala-Leu | Sodium Nitrate 100mM | X-α-D-Glucuronide |
| 91 | H7 | Propionic Acid | Ala-Thr | Sodium Nitrite 10mM | X-β-D-Glucuronide |
| 92 | H8 | Acetic Acid | Gly-Asn | Sodium Nitrite 20mM | X-β-D-Glucosaminide |
| 93 | H9 | Formic Acid | Gly-Gln | Sodium Nitrite 40mM | X-β-D-Galactosaminide |
| 94 | H10 | Aztreonam | Gly-Glu | Sodium Nitrite 60mM | X-α-D-Mannoside |
| 95 | H11 | Sodium Butyrate | Gly-Met | Sodium Nitrite 80mM | X-PO4 |
| 96 | H12 | Sodium Bromate | Met-Ala | Sodium Nitrite 100mM | X-SO4 |


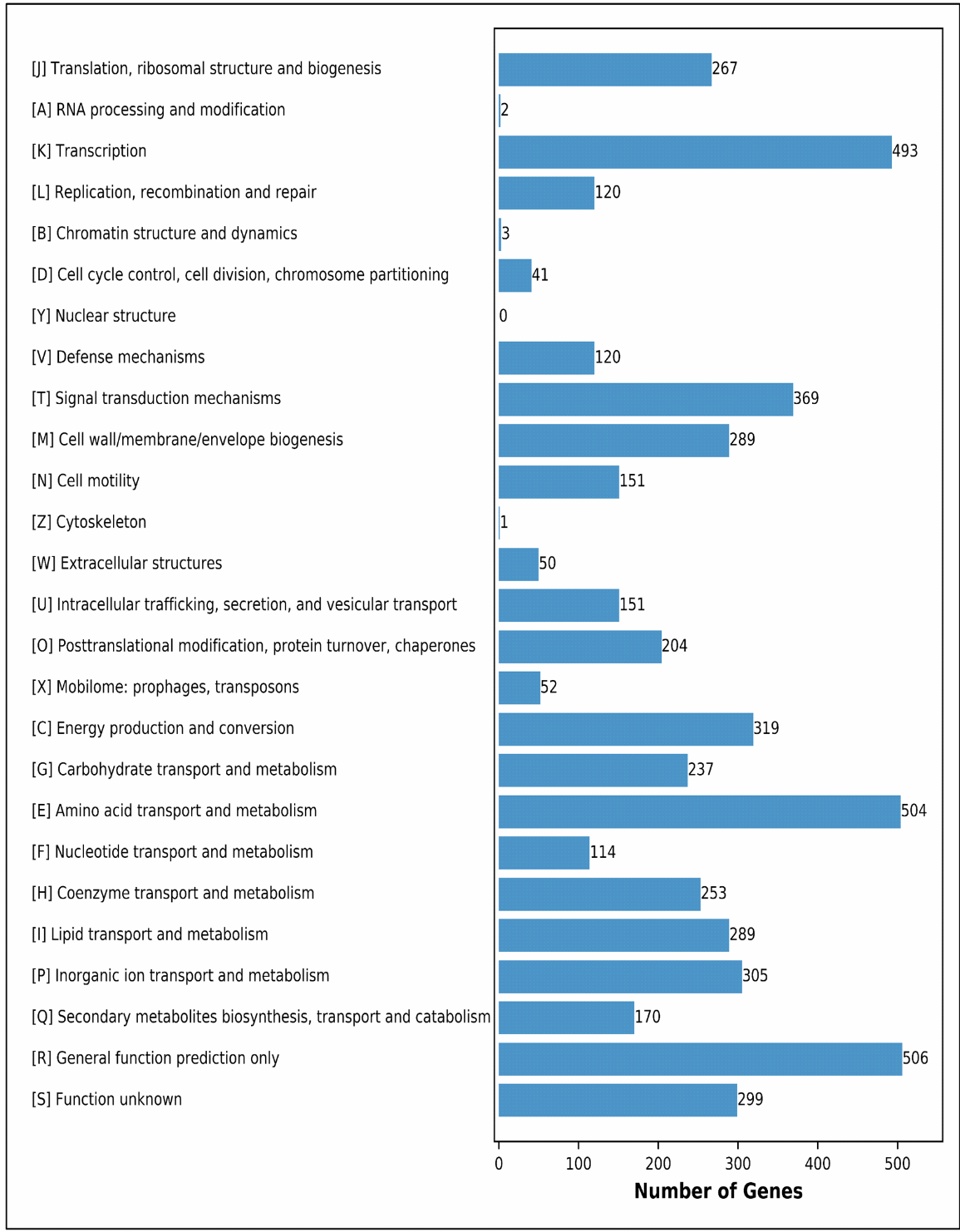


**FIGURE S1.** Functional grouping in the COG database of encoded proteins.


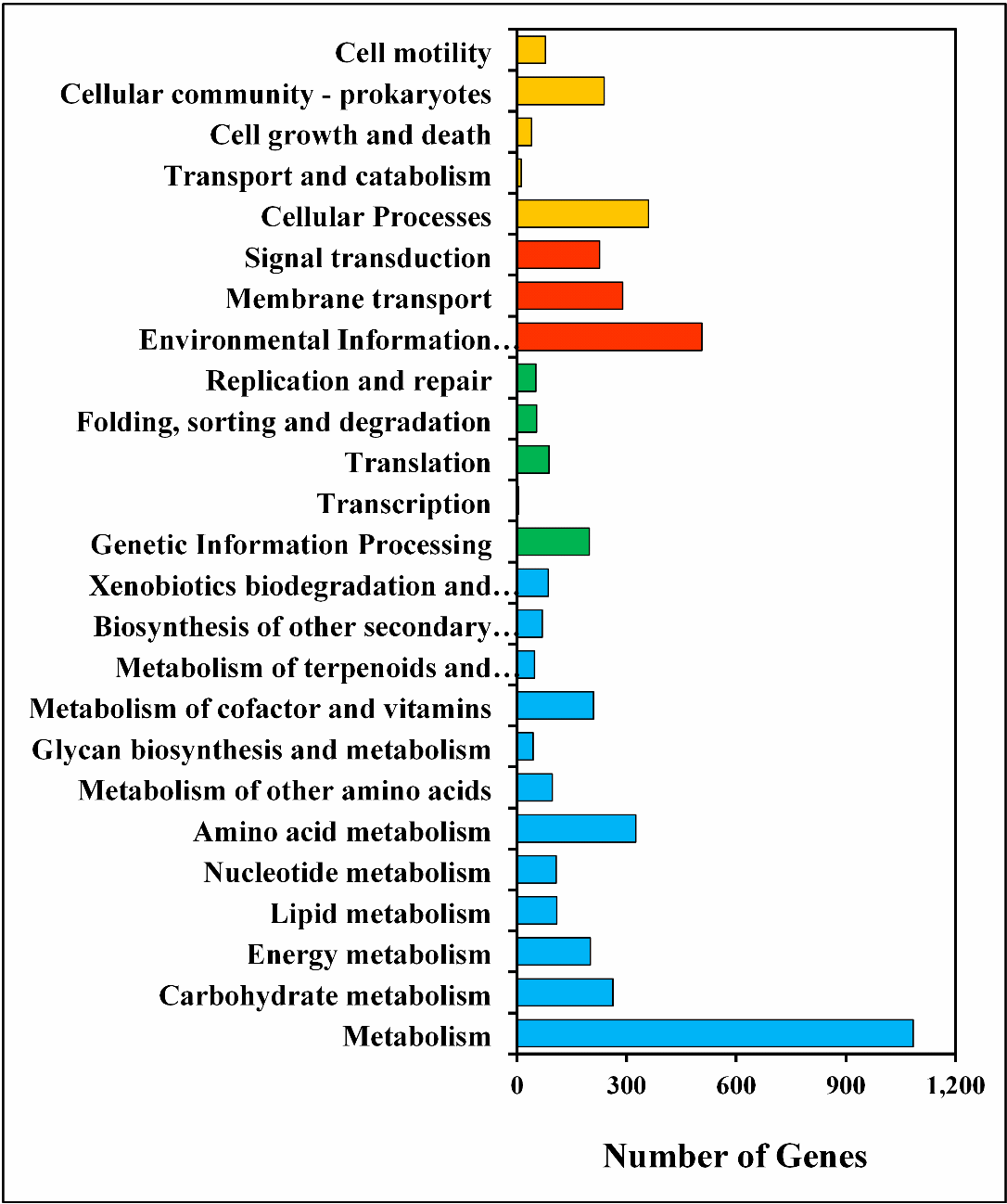


**FIGURE S2.** The functional classification of the encoded proteins in KEGG database.


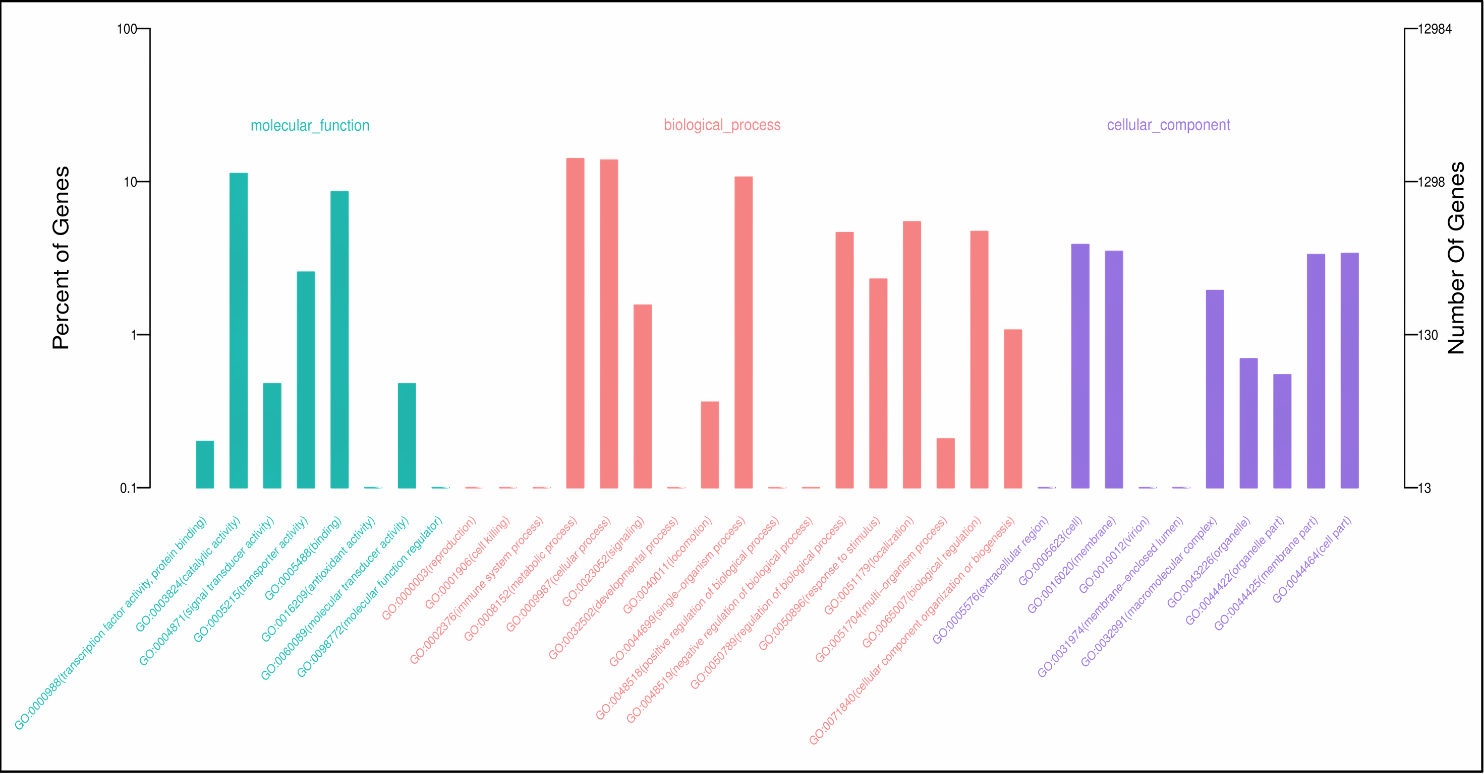


**Figure S3.** The functional classification of the encoded proteins in GO database.


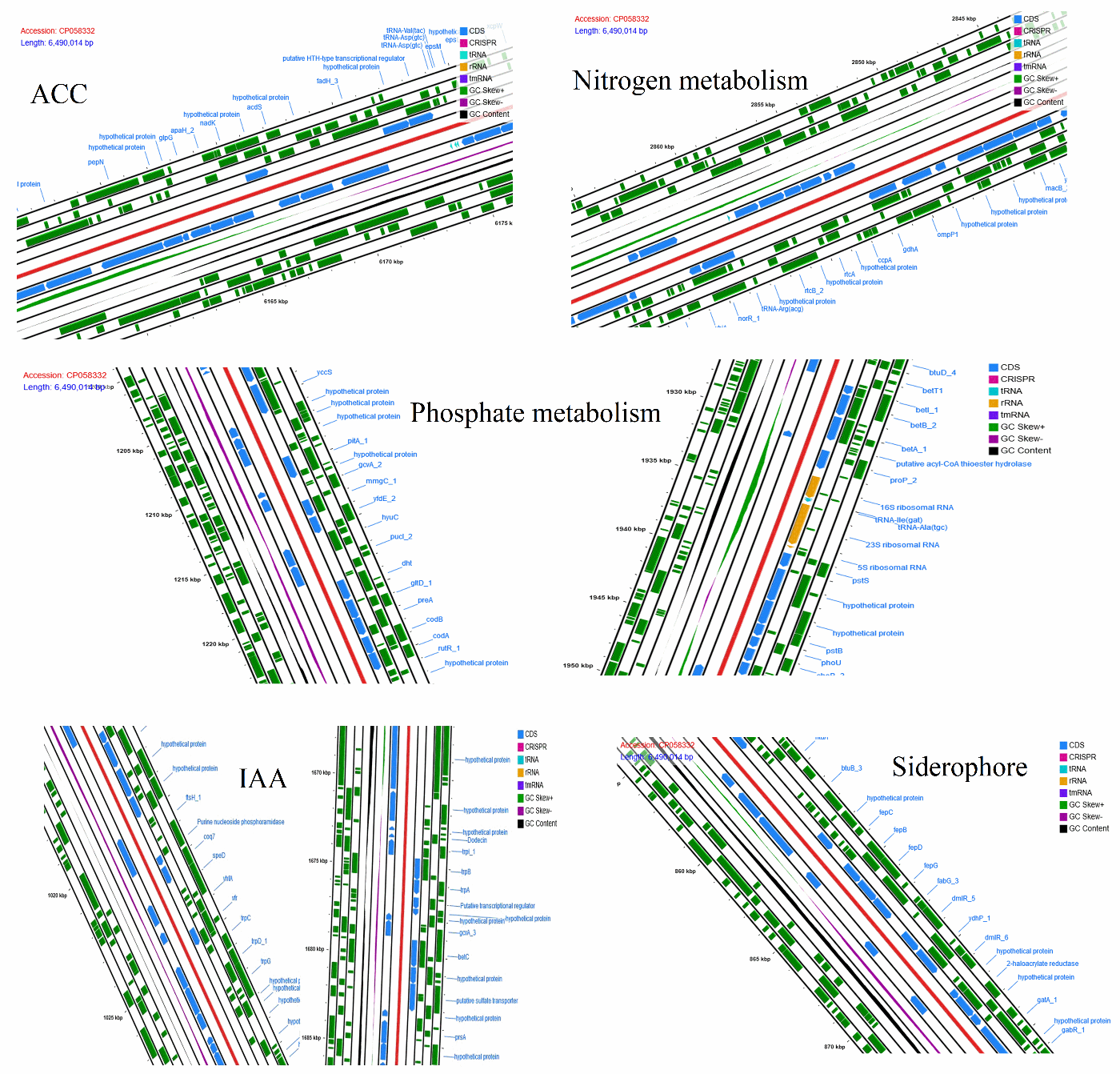


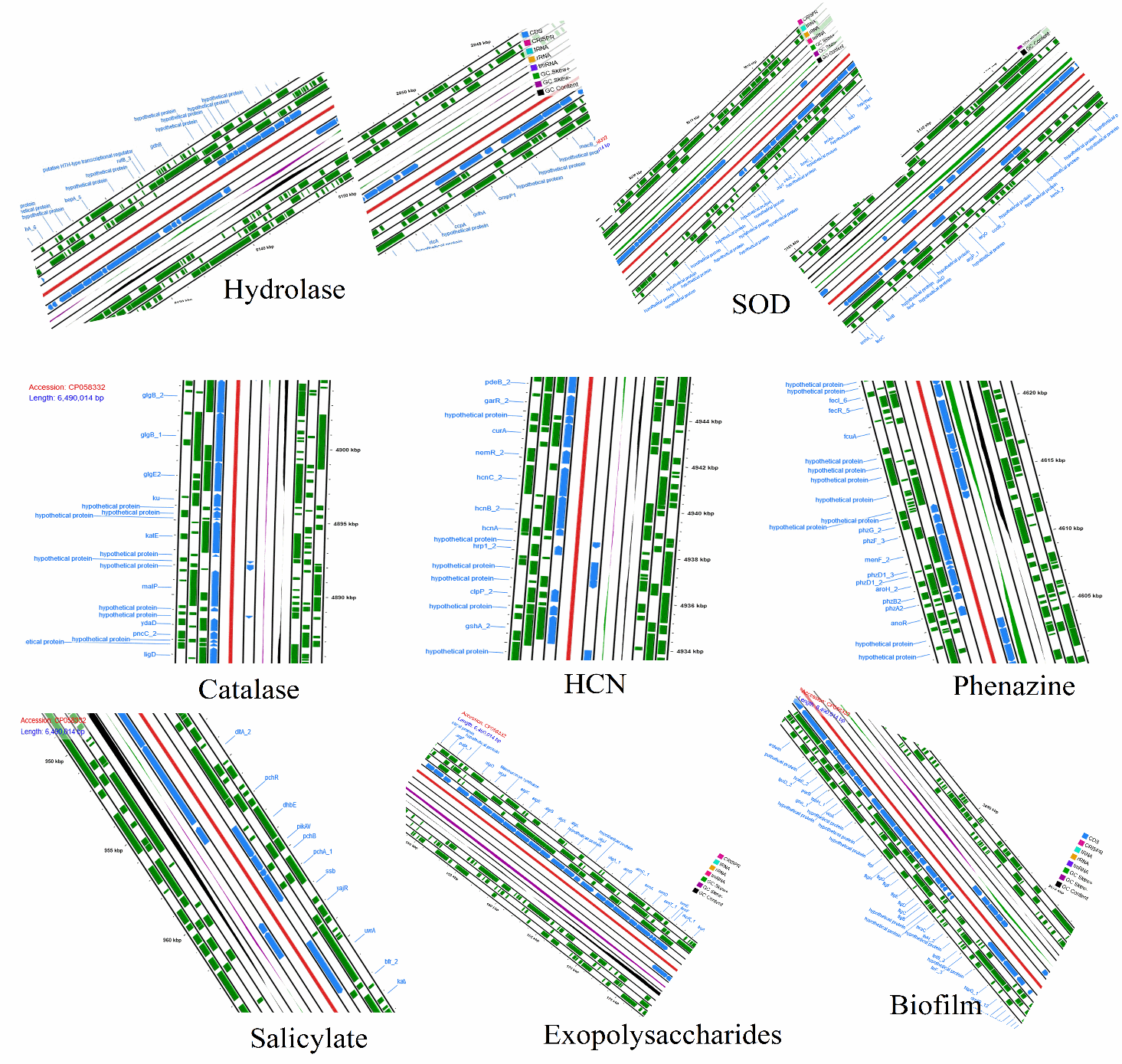


**Figure S4.** Genes associated with plant growth promotion and biocontrol activities in the genome of strain B18. ACC- 1-Aminocyclopropane-1-carboxylate deaminase, Nitrogen metabolism, Phosphate metabolism, IAA- Indole**-**3-acetic acid, Siderophores, Hydrolase, SOD- Superoxide dismutase, Catalase, HCN- Hydrogen cyanide, Phenazine, Salicylate, Exopolysaccharides, and Biofilm.


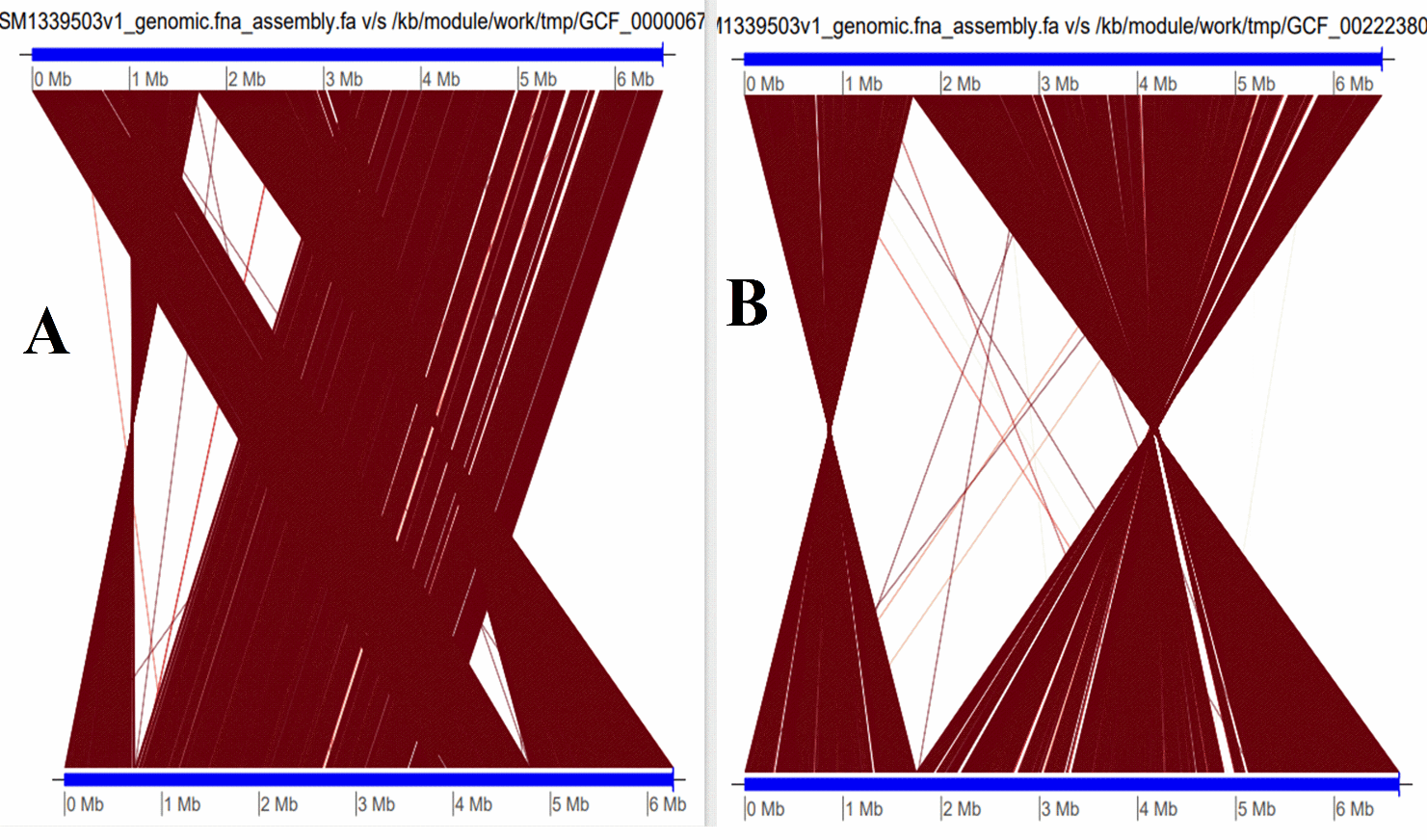


**Figure S5.** Average nucleotide identity presenting similarity between strain B18 and closely related species. The ANI value among strain B18 and *P. aeruginosa* PA01 was 99.18% and *Pseudomonas* sp. PL10 was 98.74% respectively.

**
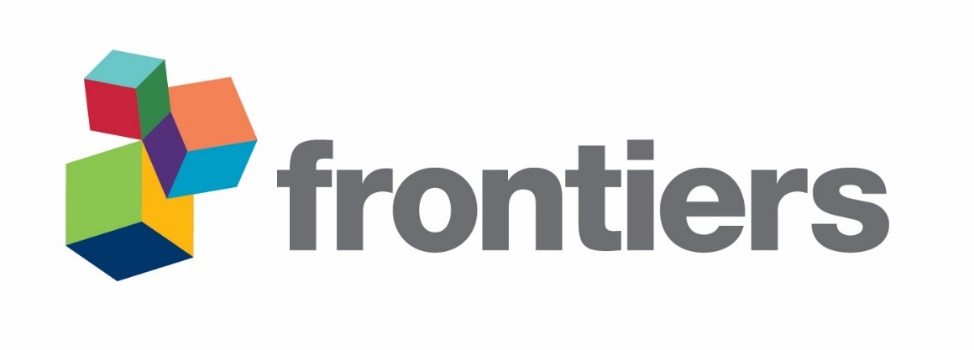
**
